# Rrp1 translocase and ubiquitin ligase activities restrict the genome destabilising effects of Rad51 in fission yeast

**DOI:** 10.1093/nar/gkab511

**Published:** 2021-06-22

**Authors:** Jakub Muraszko, Karol Kramarz, Bilge Argunhan, Kentaro Ito, Gabriela Baranowska, Yumiko Kurokawa, Yasuto Murayama, Hideo Tsubouchi, Sarah Lambert, Hiroshi Iwasaki, Dorota Dziadkowiec

**Affiliations:** Faculty of Biotechnology, University of Wrocław, Poland; Institut Curie, Université PSL, CNRS UMR3348, 91400 Orsay, France; Université Paris-Saclay, CNRS UMR3348, 91400 Orsay, France; Cell Biology Center, Institute of Innovative Research, Tokyo Institute of Technology, Japan; Cell Biology Center, Institute of Innovative Research, Tokyo Institute of Technology, Japan; Faculty of Biotechnology, University of Wrocław, Poland; Cell Biology Center, Institute of Innovative Research, Tokyo Institute of Technology, Japan; Cell Biology Center, Institute of Innovative Research, Tokyo Institute of Technology, Japan; Cell Biology Center, Institute of Innovative Research, Tokyo Institute of Technology, Japan; Institut Curie, Université PSL, CNRS UMR3348, 91400 Orsay, France; Université Paris-Saclay, CNRS UMR3348, 91400 Orsay, France; Cell Biology Center, Institute of Innovative Research, Tokyo Institute of Technology, Japan; Faculty of Biotechnology, University of Wrocław, Poland

## Abstract

Rad51 is the key protein in homologous recombination that plays important roles during DNA replication and repair. Auxiliary factors regulate Rad51 activity to facilitate productive recombination, and prevent inappropriate, untimely or excessive events, which could lead to genome instability. Previous genetic analyses identified a function for Rrp1 (a member of the Rad5/16-like group of SWI2/SNF2 translocases) in modulating Rad51 function, shared with the Rad51 mediator Swi5-Sfr1 and the Srs2 anti-recombinase. Here, we show that Rrp1 overproduction alleviates the toxicity associated with excessive Rad51 levels in a manner dependent on Rrp1 ATPase domain. Purified Rrp1 binds to DNA and has a DNA-dependent ATPase activity. Importantly, Rrp1 directly interacts with Rad51 and removes it from double-stranded DNA, confirming that Rrp1 is a translocase capable of modulating Rad51 function. Rrp1 affects Rad51 binding at centromeres. Additionally, we demonstrate *in vivo* and *in vitro* that Rrp1 possesses E3 ubiquitin ligase activity with Rad51 as a substrate, suggesting that Rrp1 regulates Rad51 in a multi-tiered fashion.

## Graphical Abstract

**Graphical Abstract:**
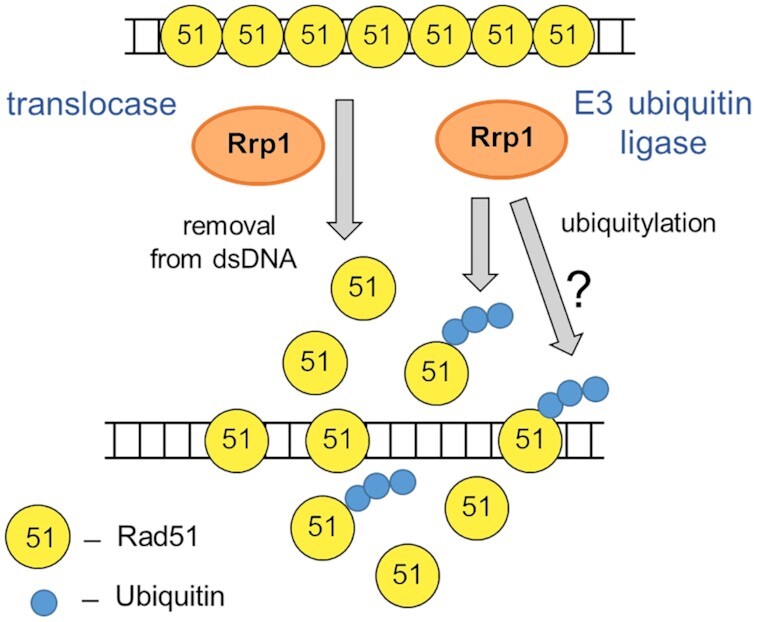
Rrp1 translocase removes Rad51 from double stranded DNA and is an E3 ubiquitin ligase with Rad51 as a substrate.

## INTRODUCTION

Homologous recombination (HR) is a highly conserved pathway for the repair of DNA double-strand breaks (DSBs), and many key HR proteins have a critical role during DNA replication (1). During DSB repair, the Rad51 recombinase forms a nucleoprotein filament on single-stranded DNA (ssDNA) that catalyses strand invasion into intact homologous double-stranded DNA (dsDNA) (2,3). Rad51 is aided by a group of proteins called recombination mediators. The main mediator in yeasts, Rad52, facilitates Rad51 loading onto replication protein A (RPA)-coated ssDNA (4,5). In human cells, the tumour suppressor protein BRCA2 fulfils an equivalent function during HR, recruiting Rad51 onto RPA-coated ssDNA and stabilising presynaptic filaments (6). Additionally, human cells contain five canonical Rad51 paralogs (RAD51B, RAD51C, RAD51D, XRCC2 and XRCC3) that influence HR, and these factors are thought to stimulate Rad51 activity, reviewed in (7). In the fission yeast *Schizosaccharomyces pombe*, three auxiliary factor complexes have been shown to promote Rad51-dependent DNA repair: Sws1–Rlp1–Rdl1, Rad55–Rad57 and Swi5–Sfr1, all of which are conserved in humans (8–11). We previously identified another complex, Rrp1–Rrp2, that acts in a Swi5–Sfr1-dependent sub-pathway of HR in the replication stress response and modulates Rad51 activity (12,13). Rrp1 and Rrp2 also have distinct roles in modulating histone dynamics that affect centromere stability (14). Additionally, Rrp2 has been shown to protect cells from Top2-induced DNA damage (15) and to play a role in telomere maintenance (14) independently of Rrp1.

Replication forks frequently stall at specific sites in the genome, such as repetitive DNA sequences, DNA lesions resulting from exogenous damage, or sites of DNA-protein association (16), and RAD51 has multiple important functions at such arrested forks. First, RAD51 binding stabilizes replication forks by protecting them from nucleolytic degradation (17,18). Second, RAD51 participates in replication fork reversal, a global mechanism to stabilise forks and protect them from breakage, and stimulates the fork regression activity of RAD54, a SWI2/SNF2-like translocase, by inhibiting fork restoration (19,20). Several SNF2-family DNA translocases, such as SMARCAL1, ZRANB3 and HLTF, are able to remodel replication forks (21–24), and RAD51 is proposed to cooperate in this process by driving the equilibrium of the reaction toward fork reversal (25). Finally, when the replication fork is inactivated or converted to a DSB by MUS81-dependent nucleolytic cleavage, the strand exchange activity of RAD51 promotes HR-dependent reconstitution of replication (26–28).

RAD51 filament formation must be tightly regulated because inappropriate, excessive, or untimely recombination (especially at replication forks or repeated sequences) can lead to deleterious effects including loss of heterozygosity and chromosome rearrangements that are hallmarks of cancer in higher organisms (29). Many helicases, such as Sgs1 and Srs2 in yeast, as well as BLM, PARI, FANCJ and RECQ5 in mammals, have been implicated in regulating the stability of RAD51 filaments formed on ssDNA. This ensures that the HR-mediated DSB repair process is reversible and can proceed along multiple pathways, making it both flexible and robust, reviewed in (30). Recently, RADX has been found to antagonise RAD51 binding to ssDNA specifically at replication forks, where it regulates the balance between RAD51 fork protection, fork reversal and its role in DSB repair (31,32).

Rad51 must not only be able to form filaments on ssDNA but also bind to dsDNA tracts in order to carry out its multiple functions, and this process is also stringently regulated. The SWI2/SNF2-like translocase Rad54 dissociates Rad51 from dsDNA in both yeast and human cells (33–35), allowing for the repair synthesis by DNA polymerases that is necessary for the completion of HR. Rad54 is activated in G2 and does not remove Rad51 from stalled replication forks (36). Another complex, MMS22L-TONSL, has been shown in human cells to limit RAD51 binding to dsDNA and stimulate HR-mediated restart of arrested replication forks (37). Importantly, other SWI2/SNF2-like translocases, namely Rdh54 and Uls1, cooperate with Rad54 in *Saccharomyces cerevisiae* not only to antagonise Rad51 binding to dsDNA during HR, but also to counteract its toxic accumulation on undamaged chromatin (38–40). RAD54L and RAD54B in humans also prevent the genome-destabilising consequences of excessive RAD51 binding to dsDNA (35). It should also be noted that the binding of RAD51 to dsDNA renders the dsDNA inaccessible to the RAD51-ssDNA filament and thus acts as a barrier to HR itself (41).

Interestingly, the RAD51 paralog RAD51C has been shown to prevent proteasomal degradation of RAD51 in human cells, especially after DNA damage (42), suggesting that RAD51 can also be regulated by ubiquitylation. Recently, RAD51 was found to be poly-ubiquitylated by the E3 ubiquitin ligase RFWD3 in a process stimulated by DNA damage (43). RAD51 ubiquitylation decreases RAD51 binding to ssDNA and leads to its proteasomal degradation, while also stimulating chromatin loading of RAD54. It has therefore been proposed that RAD51 ubiquitylation promotes its removal from sites of DNA damage and is necessary for completion of HR DNA repair (43).

Regulation of Rad51 by Fbh1, an F-box helicase and E3 ubiquitin ligase, is more complex and involves both activities of this protein. In *S. pombe*, Fbh1 acts as a translocase and disrupts Rad51 ssDNA filaments, thereby regulating the outcome of the HR reaction. Additionally, the SCF^Fbh1^ E3 complex can ubiquitylate Rad51 *in vitro* and this modification was found to be necessary for depletion of Rad51 in stationary-phase cells (44). Human RAD51 is also monoubiquitylated *in vitro* by SCF^FBH1^ but this does not result in the protein's turnover by proteolysis (45). Instead, the authors propose that during replication stress, FBH1 translocase displaces RAD51 from ssDNA and modifies it to prevent its reloading, thus restricting untimely HR at the replication fork.

Many proteins with helicase, translocase and/or ubiquitin ligase activities have been found to regulate Rad51 activity, underscoring the importance of this process. Here, we show that *S. pombe* Rrp1, an orthologue of *S. cerevisiae* Uls1, belonging to the unique RING-domain-containing Rad5/16-like group of SWI2/SNF2 translocases, affects Rad51 binding at centromeres, ubiquitylates Rad51 and is able to displace it from dsDNA. We propose that these translocase and ubiquitin ligase activities allow Rrp1 to counteract the genotoxic effects of excessive Rad51 binding to specific regions of chromatin, such as centromeric regions.

## MATERIALS AND METHODS

### Yeast strains, plasmids and general methods

Strains, plasmids and primers used are listed in Supplementary Tables S1, S2 and S3, respectively.

Media used for *S. pombe* growth were as described (46). Yeast cells were grown at 28°C in complete yeast extract plus supplements (YES) medium or glutamate supplemented Edinburgh minimal medium (EMM). Where required, thiamine was added at 5 μg/ml and geneticin (ICN Biomedicals) at 100 μg/ml. Strains were obtained by classical yeast genetics techniques. pREP81-FLAG vector and plasmids carrying wild-type and mutated forms of *rrp1*^+^ and *rrp2*^+^, as well as domains of the *rrp1*^+^ gene used in the yeast two hybrid system, were constructed using the Gibson Assembly^®^ Cloning Kit (NEB). Amplified fragments were cloned into NdeI and BamHI digested pREP81 vector. After Gibson cloning, inserts were cut by NdeI and SmaI digestion and cloned into pREP42-HA, pREP42-EGFP, pREP41-mCherry, or pGADT7 and pGBKT7 plasmids. *rrp1*^+^ and *rrp1-CS* mutant version were introduced into the pGEX vector (GE Healthcare) by In-Fusion^®^ cloning (Takara Bio). All constructs were checked by sequencing.

### Spot assays

Cells were grown to mid-log phase, then serially diluted 10-fold, and 2 μl aliquots were spotted onto relevant plates (YES or EMM) that were incubated for 3–5 days at 28°C and photographed. All assays were repeated at least twice.

### Survival assay

Cells were grown for 48 h in YES or in minimal medium with (repressed conditions) or without thiamine (overexpression) at 28°C. 500 μl aliquots were collected, serially diluted, plated onto YES plates and incubated for 3–5 days at 28°C. The viable cells were counted and percentage of survival for gene overexpression conditions was calculated against the repressed control.

### Yeast two-hybrid assay

Gal4-based Matchmaker Two-Hybrid System 3 (Clontech) was used. The indicated proteins were fused to the GAL4 activation domain (AD) in pGADT7 vector and the GAL4 DNA-binding domain (DBD) in pGBKT7, and expressed in the *S. cerevisiae* tester strain AH109. Transformants were selected on synthetic dextrose drop-out medium without Leu and Trp (SD DO-2), and then plated on low stringency medium without Leu, Trp and His (SD DO-3) and high stringency medium without Leu, Trp, His and Ade (SD DO-4), and incubated for 3–5 days at 28°C.

### Fluorescence microscopy

To determine the formation of Rrp1 foci and their co-localisation with Rad51 foci, appropriate transformants were grown for 24 h in EMM medium without thiamine. 1 ml of culture was harvested, washed with water and subjected to fluorescent microscopy analysis. For co-localisation experiments, data were collected under 63× magnification with the confocal microscope Leica 453 TCS SP8 (Leica Microsystems) equipped with Leica HyD SP detector, and analysed with LAS X 3.3.0. For examination of mitotic defects induced by *rad51*^+^ overexpression, samples taken from respective transformant cultures grown for 48 h in EMM medium without thiamine were washed and fixed in 70% ethanol. After rehydration, cells were stained with 1 mg/ml 4′,6-diamidino-2-phenylindole (DAPI) and 1 mg/ml *p*-phenylenediamine in 50% glycerol and examined by fluorescence microscopy with Axio Imager A.2 (Carl Zeiss).

### Rad51 foci detection by immunofluorescence

Strains grown in complete EMM media were fixed with formaldehyde at 4% final concentration for 45 min with shaking at room temperature then washed with PBS and subsequently with PEM buffer (100 mM PIPES, 1 mM EGTA, 1 mM MgSO_4_, pH 6.9). Next, the cell wall was digested for 10 min at 30°C with 100T zymolyase (MP Biomedicals, SKU08320932) at a final concentration of 0.5 μg/ml in PEMS (PEM with 1.2 M sorbitol). After three washes with PEMS, cells were treated with 1% triton X-100 in PEMS for 5 min at RT. Next, cells were washed twice with PEMBAL (1% BSA, 0.1% sodium azide, 100 mM lysine monohydrate (Sigma L-5626) in PEM buffer) and incubated on the wheel for 1 h in PEMBAL. Subsequently, cells were resuspended in 300 μl of PEMBAL with anti-Rad51 antibody (Abcam, ab63799, 1:300) overnight on a wheel at room temperature. After washes with PEMBAL buffer, two of 10 min each and a third for 30 min on a wheel, the cells were resuspended in 300 μl of PEMBAL with anti-Rabbit Alexa Fluor 555 (Molecular Probes, A21428, 1:1000) for 2 h at room temperature. After three washes in PBS, each for 10 min, cells were resuspended in 1 ml of PBS with DAPI (diluted 1:4000) for 3 min. Then cells were washed with PBS to remove excess DAPI. Finally, cells were resuspended in 10 μl of ProLong Gold antifade reagent (Invitrogen, P36934) and subjected to snapshot microscopy on glass slides using a 3D LEICA DMRXA microscope, supplied with CoolSNAP monochromic camera (Roper Scientific) under 100× oil immersion magnification with numerical aperture 1.4. In all, 14 z-stack pictures of 300 nm with 400 ms exposure for Alexa 555 and 50 ms for DAPI channels were collected with METAMORPH and analyzed with ImageJ Software.

### Chromatin immunoprecipitation (ChIP) of Rad51

ChIP against Rad51 was performed as described in (47) with the following modifications. 100 ml of logarithmic cell cultures with thiamine (*RTS1*-RFB OFF, inactive replication fork block on chromosome III) or without thiamine (*RTS1*-RFB ON, active replication fork block on chromosome III) were crosslinked with 10 mM DMA (dimethyl adipimidate, Thermo Scientific, 20660) and then with 1% formaldehyde (Sigma, F-8775). Next, cells were frozen in liquid nitrogen and lysed by bead beating in 400 μl of lysis buffer (50 mM HEPES pH 7.5, 1% Triton X100, 0.1% sodium deoxycholate, 1 mM EDTA, 1 mM PMSF and Complete EDTA-free protease inhibitor cocktail tablets (Roche, 187358). After that, chromatin sonication was performed using Diagenode Bioruptor, 10 cycles of 30 s ON and 30 s OFF at 4°C. Immunoprecipitation was done overnight with an anti-Rad51 antibody (Abcam, ab63799, 1:300). Protein G Dynabeads (Invitrogen, 10003D) were then added for 1 h and crosslinking was reversed by incubating the sample at 65°C for 2 h. DNA associated with Rad51 was purified with Qiaquick PCR purification kit (QUIAGEN, 28104) and eluted in 400 μl of water. qPCR (iQ SYBR green supermix, Biorad, 1708882, primers listed in Supplementary Table S3) was performed to determine the relative amounts of DNA (starting quantities based on standard curves for each pair of primers) using BIORAD CFX Maestro v1.1. The enrichment at each locus was determined by subtracting the negative control values (*rad51*Δ strain) and internal control locus at chromosome II (named II.50).

### *In vivo* co-immunoprecipitation

*In vivo* pull-down experiments were performed using strains with native levels of Rad51 and overproduction of FLAG-tagged Rrp1 or Rrp2 under the control of the low strength *nmt81* promotor. Cells were grown to mid-log phase using EMM minimal medium for 24 h. 100 ml of cells were harvested and broken with glass beads in H buffer (50 mM HEPES–KOH pH 7, 50 mM KOAc, 5 mM MgOAc, 0.1% NP-40, 10% glycerol, 1 mM DTT and 1× cOmplete™ EDTA-free protease inhibitor cocktail (Roche)). Extracts were cleared by centrifugation and immunoprecipitated with ANTI-FLAG^®^ M2 Affinity Gel (Sigma). Beads were washed and eluted using 100 μg/ml 3xFLAG peptide (Sigma). For detection, anti-FLAG (1:5000, Sigma) antibodies and anti-Rad51 (1:5000, (48)) antiserum were used.

### Purification of Rrp1-FLAG

Recombinant Rrp1 and Rrp1-CS were expressed in the Rosetta *E. coli* strain (Novagen) from the pGEX-6P plasmid (GE Healthcare). Proteins were C-terminally fused to the GST tag and N-terminally to the 3xFLAG tag; only the former was removed during the purification process. Expression was induced with 1 mM IPTG (Sigma) at 18°C overnight. Cells were collected by centrifugation, resuspended in R buffer (20 mM Tris, pH 7.5, 10% glycerol, 1mM EDTA) containing 500 mM NaCl, and disrupted by sonication. The cell lysate was then clarified by ultracentrifugation (70 000 *g*, 1 h, 2°C). The supernatant was mixed with 4B GSH sepharose (Sigma) for 3 h at 4°C. Resin-bound proteins were eluted in R buffer containing 300 mM NaCl and 40 mM glutathione (Sigma). The sample was supplemented with 0.2 μg/ml of HRV-3C protease (Sigma) to remove the GST tag and dialyzed against R buffer containing 100 mM NaCl (overnight, 4°C). The dialyzed sample was loaded onto a 1 ml HiTrap Heparin (Sigma) column. Rrp1 eluted at around 650 mM NaCl with a linear gradient of 0.1–1.0 M NaCl in R buffer. Eluted fractions were diluted 6.5-fold with R buffer and loaded onto a 1 ml Resource Q column (GE Healthcare). Rrp1 eluted at around 300 mM NaCl with a linear gradient of 0.1–1.0 M NaCl in R buffer. Eluted fractions were diluted 3-fold with R buffer and loaded onto a HiTrap SP column (GE Healthcare). Rrp1 eluted at around 600 mM NaCl with a linear gradient of 0.1–1.0 M NaCl in R buffer. Eluted fractions were collected and dialysed against R buffer containing 200 mM NaCl. Concentration was determined using NanoDrop (ThermoFisher) with a molar extinction coefficient of 100 365. For the Rrp1-CS mutant, Resource Q and HiTrap SP columns were omitted. Rad51 was purified exactly as previously described (49). Uba1 (E1) and Ubc4 (E2) were purified exactly as previously described (44). All proteins were free of nuclease and/or protease activities for the duration of the relevant assays.

### *In vitro* co-immunoprecipitation

*in vitro* co-immunoprecipitation assays were performed as follows. Briefly, purified Rad51 and FLAG-tagged Rrp1 (250 nM each) were incubated in IP buffer (35 mM Tris–HCl pH 7.5, 1 mM DTT, 100 mM NaCl, 3.5 mM MgCl_2_, 0.1% NP-40, 5% glycerol) for 30 min at 30°C. Proteins were immunoprecipitated using ANTI-FLAG^®^ M2 Affinity Gel (Sigma) for 2 h at 4°C. Beads were washed three times with IP buffer, and proteins were eluted using 100 μg/ml 3x FLAG peptide (Sigma). Eluates were analysed by western blotting with anti-FLAG antibodies (1:5000, Sigma) and anti-Rad51 (1:5000, (48)) antiserum.

### Colorimetric ATPase assay

Reaction mixtures (22.5 μl) in ATPase buffer (25 mM Tris–HCl [pH 7.5], 1 mM DTT, 20 mM NaCl, 5 mM MgCl_2_ and 2% glycerol) with 30 nM Rrp1 and containing 5 μM nt ssDNA, 10 μM nt dsDNA or no DNA were prepared on ice. The reactions were initiated by the addition of 2.5 μl of 10 mM ATP (final concentration of 1 mM) and incubated at 30°C. Aliquots (10 μl) taken at time 0 and 15 min were mixed with 2 μl of 120 mM EDTA to stop the reaction. Inorganic phosphate generated by ATP hydrolysis was detected using the Malachite Green Phosphate Assay Kit (BioAssay Systems, USA). The ssDNA used in this assay was 16A(–), an 83-mer oligo (5′-AAATGAACAT AAAGTAAATA AGTATAAGGA TAATACAAAA TAAGTAAATG AATAAACATA GAAAATAAAG TAAAGGATAT AAA-3′) (50). The dsDNA used was prepared by annealing of 16A(–) and its complementary 83-mer ssDNA, 16A(+).

### Electrophoretic mobility shift assay (EMSA)

Purified Rrp1 was incubated with cssDNA (circular single-strand DNA of Phi X174 viral DNA, NEB), ldsDNA (linear double-stranded DNA obtained by digesting Phi 174 RF I with ApaLI, NEB) or cccDNA (covalently closed-circular DNA of Phi 174 RF I, NEB) at 0.5 μM (base pair concentration) in E buffer (25 mM HEPES pH 7.5, 1 mM DTT, 60 mM KCl, 2 mM ATP, 3.5 mM MgCl_2_, 5% glycerol) for 15 min in 37°C. Samples were then crosslinked with 0.2% glutaraldehyde (37°C, 5 min) then run on a 0.8% agarose 1xTAE gel and stained with SYBR Gold (ThermoFisher).

### *In vitro* ubiquitylation assay

Reactions were performed in ubiquitylation buffer (25 mM Tris–HCl [pH 7.5], 1 mM DTT, 20 mM NaCl, 5 mM ATP, 5 mM MgCl_2_ and 2% glycerol) containing 5 μM *S. cerevisiae* Ubiquitin (Funakoshi, U-100SC-05M), 0.2 μM His-Uba1 (E1), 2 μM His-Ubc4 (E2), 0.2 μM Rrp1-FLAG (E3) and 1 μM Rad51. This mixture was incubated at 37°C for 30 min. When the effect of DNA was examined, 1 μM Rad51 was preincubated with increasing concentrations of ldsDNA (PhiX RFI linearised with ApaLI) at 37°C for 10 min in ubiquitylation buffer supplemented with 50 mM NaCl prior to the addition of ubiquitylation reaction components. Reactions were stopped by addition of 6× SDS Laemmli buffer and proteins were resolved by 15% SDS-PAGE and analysed by western blotting with anti-ubiquitin antibodies (1:2000 Abcam P4D1) and anti-Rad51 (1:5000) (48) antiserum.

### Rad51 removal from DNA

EMSA-based analysis was performed as described above except that Rad51 was first incubated with the DNA at 37°C for 5 min, then Rrp1 was added. For the measurements with ssDNA, Rad51 filaments were formed by incubation of 0.5 μM Rad51 with 1.5 μM ssDNA (oligo-dT, 72-mer, base pair concentration) for 5 min at 37°C in buffer (30 mM HEPES pH 7.5, 1 mM DTT, 50 mM NaCl, 100 mM KCl, 2 mM ATP, 8 mM PC, 8 U/ml CPK, 3.5 mM MgCl_2_, 2.5% glycerol). This mixture was transferred to a 0.2 × 1.0 cm cuvette (Hellma Analytics) at 37°C. The change in fluorescence anisotropy at 575 nm following excitation at 546 nm was measured for 60 s. After that time, scavenger ssDNA (15 μM nucleotides PhiX 174 virion DNA) and the indicated concentrations of Rrp1 protein were added. Data were collected using an FP-8300 spectrofluorometer (JASCO) every second for 3 min. For each reaction, the measurements 20 s before addition of scavenger DNA and Rrp1 were averaged, and the subsequent measurements were expressed relative to this averaged value.

For the measurements with dsDNA, 6 μM of Rad51 was incubated with 3 μM (base pair concentration) of dsDNA (5′-TAMRA-labeled 16A(–) annealed to 16A(+)) for 5 min at 37°C in buffer (30 mM HEPES pH 7.5, 1 mM DTT, 50 mM NaCl, 100 mM KCl, 2 mM ATP, 8 mM PC, 8 U/ml CPK, 3.5 mM MgCl_2_, 2.5% glycerol). This mixture was then transferred to a 1.0 × 1.0 cm cuvette (Hellma Analytics) with a magnetic stirrer and maintained at 37°C with stirring (450 rpm). The change in fluorescence anisotropy at 575 nm following excitation at 546 nm was monitored for 60 s. After that time, scavenger ssDNA (15 μM nucleotides PhiX 174 virion DNA) was added, and after 60 s, 0.25 μM of Rrp1 was injected into the mixture. Data were collected using an FP-8300 spectrofluorometer (JASCO) every second for over 500 s.

Δ anisotropy was calculated using the following equation:

$$\begin{eqnarray*} && \Delta\, {\rm anisotropy} \\ && \,\, = {\rm raw}\, {\rm value}\, {\rm of}\,({\rm anisotropy}\, {\rm with}\, {\rm proteins}\, {\rm at}\, {\rm time}\, t) \\ && \,\,- ({\rm anisotropy}\, {\rm without}\, {\rm proteins}\, {\rm at}\, {\rm time}\, t) \end{eqnarray*}$$

Time zero was defined as the time when Rrp1 was added into the reaction mixture.

The dissociation rate (*k*_off_) values were obtained by fitting the time dependence of Δ anisotropy, with the following equation:

$$\begin{eqnarray*} \Delta\, {\rm anisotropy} &=& ({\rm Maximum}\, {\rm value}\, {\rm of}\, \Delta \,{\rm anisotropy}) \\ && \times\, {\rm exp} ({-k_{off}} \cdot t) \end{eqnarray*}$$

### Statistical data analysis

For viability assays ANOVA test, and for ChIP assays two-sided Student's *t*-test, were used to calculate the *P*-values. To assess statistical significance of proportions of cells with aberrant mitosis and the Rad51 localisation pattern, the *Z*-test for two population proportions was used to calculate the *z*-statistic and corresponding *P*-values. (*** *P* ≤ 0.001, ** 0.001 < *P* ≤ 0.01, * 0.01 < *P* ≤ 0.05).

## RESULTS

### Rrp1 counteracts *rad51*^+^ overexpression-induced toxicity

**Figure 1.**
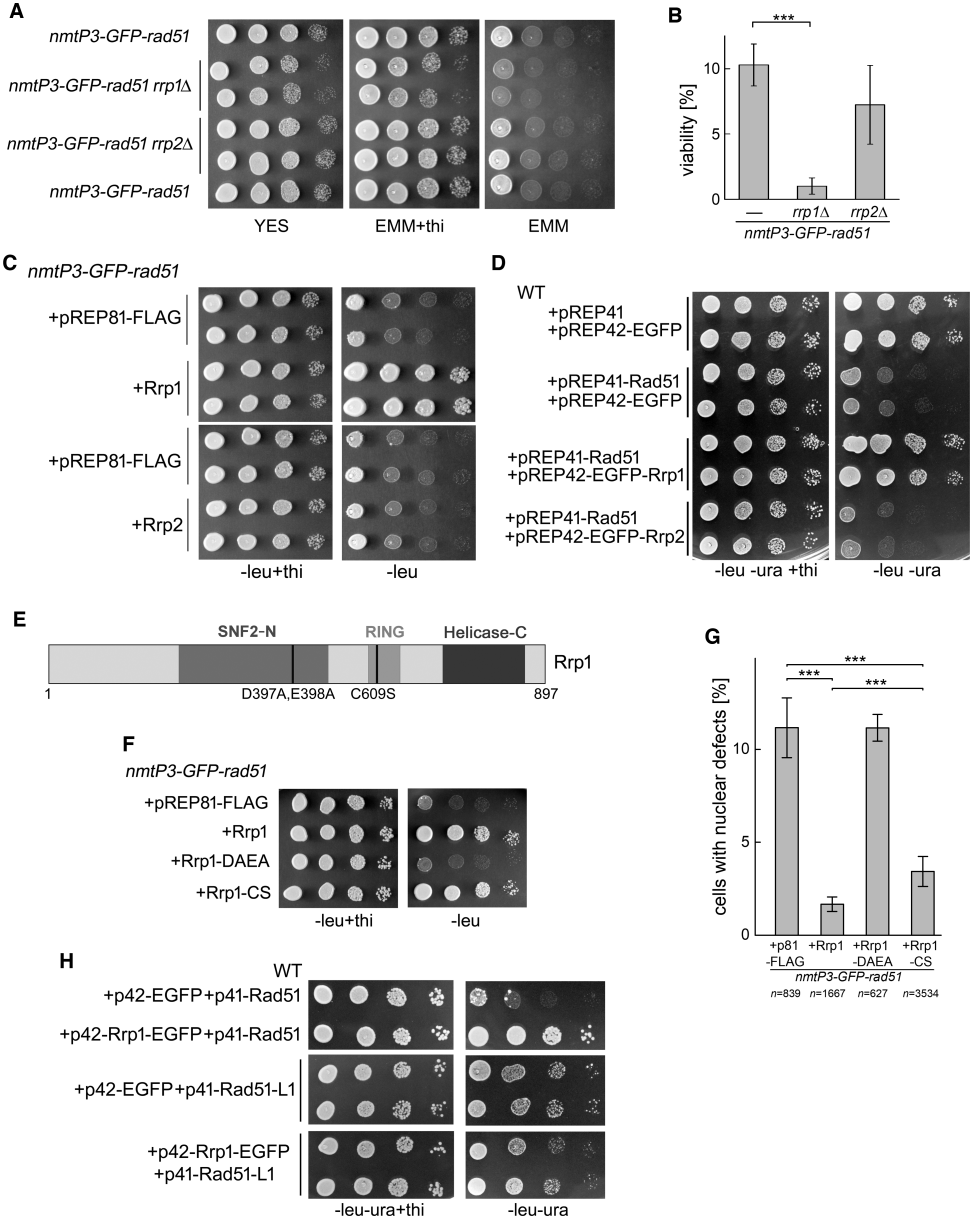
The ATPase activity of Rrp1 is important for viability and proper chromosome segregation in cells overexpressing *rad51*^+^. (**A**) Deletion of *rrp1*^+^, but not *rrp2*^+^, exacerbates the growth defect induced by *GFP-rad51*^+^ overexpression. Growth of the indicated strains under conditions where *GFP-rad51*^+^ overexpression was induced (EMM) or repressed (EMM +thiamine, YES control plate) was examined by spot test analysis. (**B**) Viability loss caused by *GFP-rad51*^+^ overexpression is greater in *rrp1*Δ cells. Ratio of surviving cells for strains grown in the absence of thiamine (induction of expression) to those grown in its presence (without induction) was determined. The experiment was repeated for four independent cultures of each strain. ANOVA test was used to calculate *P-*values. (**C**) Spot test analysis demonstrating that overexpression of *rrp1*^+^, but not *rrp2*^+^, suppresses the growth defect caused by GFP-Rad51 overproduction (-leu). (**D**) GFP tag is not responsible for the growth defect seen in GFP-Rad51 overproducing cells and simultaneous overexpression of *rrp1*^+^, but not *rrp2*^+^, suppresses this toxicity. Wild-type cells were transformed with plasmids containing genes for Rrp1 or Rrp2 and untagged Rad51, to assess by spot test analysis the effect of their overexpression on growth (-leu-ura). Two independent transformants for each set of plasmids are shown. (**E**) Mutations abolishing Rrp1 putative SWI2/SNF2 DNA translocase (Walker B mutant: DAEA) and ubiquitin ligase (RING mutant: CS) activity are shown. (**F**) Functional Rrp1 translocase is required for suppression of the growth defect in *GFP-rad51*^+^ overexpressing cells. Cells were transformed with plasmids harbouring genes for wild-type or mutated versions of the respective proteins and the effect of their overexpression on the growth of cells overproducing GFP-Rad51 (leu) or not (–leu+thi) was assessed by spot test analysis. (**G**) Accumulation of mitotic aberrations in *GFP-rad51*^+^ overexpressing cells is prevented by *rrp1*^+^ overexpression and depends on the functional translocase, but not the RING domain. Cells with unequally segregated genetic material (cut and non-disjunction) were observed by DAPI staining of the nuclei of transformants grown for 48 h in media lacking thiamine. *n* = total number of cells counted for 3 independent transformants of vector, *rrp1*^+^, *rrp1-DAEA or rrp1-CS*. The error bars represent the standard deviation about the mean values. The Z-test for two population proportions was used to calculate the *Z*-statistic and corresponding *P*-values. (**H**) Overproduction of the Rad51-L1 (F254A) mutant defective in DNA binding results in only a mild growth defect that is not rescued by overproduction of Rrp1. Wild-type cells were transformed with plasmids harbouring genes for Rrp1 and wild-type Rad51 or Rad51-L1, and the effect of their overexpression on growth (leu-ura) was assessed by spot test analysis. Two independent transformants overproducing Rad51-L1 are shown. (*** *P* ≤ 0.001, ** 0.001 < *P* ≤ 0.01, * 0.01 < *P* ≤ 0.05).

Previous studies have shown that *rad51*^+^ overexpression in *S. pombe* results in a severe growth defect (51). Rad51 overproduction leads to its excessive accumulation on chromatin and has a negative effect on cell growth and chromosome stability that is aggravated in mutants devoid of SWI2/SNF2-related translocases: RAD54L and RAD54B in humans (35), and Rdh54, Rad54 and Uls1 in *S. cerevisiae* (39). Two *ULS1* orthologues, Rrp1 and Rrp2, have been identified in *S. pombe* (12), so we set out to examine their functional interaction with Rad51. Using an *nmtP3-GFP-rad51* strain, where the *GFP-rad51*^+^ gene is expressed from a strong *nmt* promoter (*nmtP3*) that is induced in media lacking thiamine, we confirmed that induction of *rad51*^+^ expression (Figure 1A, EMM plate) resulted in a severe growth defect and loss of viability (Figure 1B). Importantly, this was further exacerbated by deletion of *rrp1*^+^, but not *rrp2*^+^. Growth inhibition of the *nmtP3-GFP-rad51 rrp1*Δ strain was visible even on media where gene expression from the *nmtP3* promoter is very limited (Figure 1A, YES and EMM+thi plates), indicating that even a mild increase in Rad51 protein levels may be toxic when Rrp1 is absent.

We thus hypothesised that if deletion of *rrp1*^+^ is toxic in the *nmtP3-GFP-rad51* strain, *rrp1*^+^ overexpression should be beneficial. Indeed, we found that overexpression of *rrp1*^+^, but not *rrp2*^+^, from the low-strength *nmt* promoter (*nmt81*), rescued the growth defect (Figure 1C) and viability loss induced by GFP-Rad51 overproduction (Supplementary Figure S1A). The level of Rrp1 and Rrp2 proteins in these cells was comparable (Supplementary Figure S1B).

Overproduction of Rad51 without the GFP tag resulted in similar toxicity as GFP-Rad51 and simultaneuos overproduction of Rrp1, but not Rrp2, rescued the associated growth defect (Figure 1D) and viability loss (Supplementary Figure S1C) to the wild-type level (all genes were overexpressed from the medium-strength *nmt* promoters *nmt41* or *nmt42*). These results indicate that the observed phenomena are not caused by the GFP tag on Rad51, allowing us to utilise a synthetic dosage lethality approach with the *nmtP3-GFP-rad51* strain to assess the effect of Rrp1 on Rad51 activity.

### The ATPase activity of Rrp1 is required to counter the genotoxicity associated with Rad51 overproduction

Rrp1 shares a complex domain structure with Uls1 (12), and contains an ATPase domain with Walker A and B motifs, characteristic for SNF2 translocases, as well as a RING domain typical for ubiquitin ligases. Walker B (*rrp1-DAEA*) and RING (*rrp1-CS*) Rrp1 mutant variants (Figure 1E) were therefore used to examine the importance of the putative DNA translocase and ubiquitin ligase Rrp1 activities for counteracting *GFP-rad51*^+^ overexpression-induced toxicity. While the presence of a functional Rrp1 ATPase domain is required for restoration of normal growth to the *GFP-rad51^+^* overexpressing strain, the RING domain appears to be dispensable (Figure 1F). The protein levels of Rrp1, Rrp1-DAEA and Rrp1-CS in these cells were comparable (Supplementary Figure S1D).

It has previously been shown that *rad51*^+^ overexpression results in mitotic aberrations revealed by the accumulation of cells with nuclei exhibiting the cut (*cell untimely torn*) phenotype (51). We demonstrated that overproduction of Rrp1, but not the Rrp1-DAEA mutant, was able to suppress the appearance of nuclear defects, such as cut and non-disjunction, that occur in the *GFP-rad51*^+^ overexpressing strain (Figure 1G). Interestingly, the rescue induced by Rrp1-CS overproduction was less pronounced than that by wild-type Rrp1, with more cells undergoing aberrant mitosis (Figure 1G). Thus, replication stress in *rad51*^+^ over-expressing cells was greater when Rrp1-CS, rather than wild-type Rrp1, was overproduced although it did not reach a level that would cause a growth defect (Figure 1F). This suggests that Rrp1 ubiquitin ligase activity might also have a role in the protein's functional interaction with Rad51.

It has been proposed that excessive Rad51 binding to undamaged DNA is the cause of Rad51 overproduction toxicity in human cells (35). Indeed, we found that overproduction of the Rad51-L1 (F254A) mutant, which corresponds to human RAD51-L1 (Y232A) that is defective in DNA binding (52), resulted in only slight growth inhibition, and *rrp1*+ overexpression did not affect this phenotype (Figure 1H). This was not due to the lower level of Rad51-L1 than wild-type Rad51 produced from the constructs used (Supplementary Figure S1E). Taken together, these data imply that Rrp1 may counteract Rad51-overproduction induced toxicity by interfering with Rad51 binding to DNA.

### Rrp1 prevents excessive Rad51 accumulation on DNA

**Figure 2.**
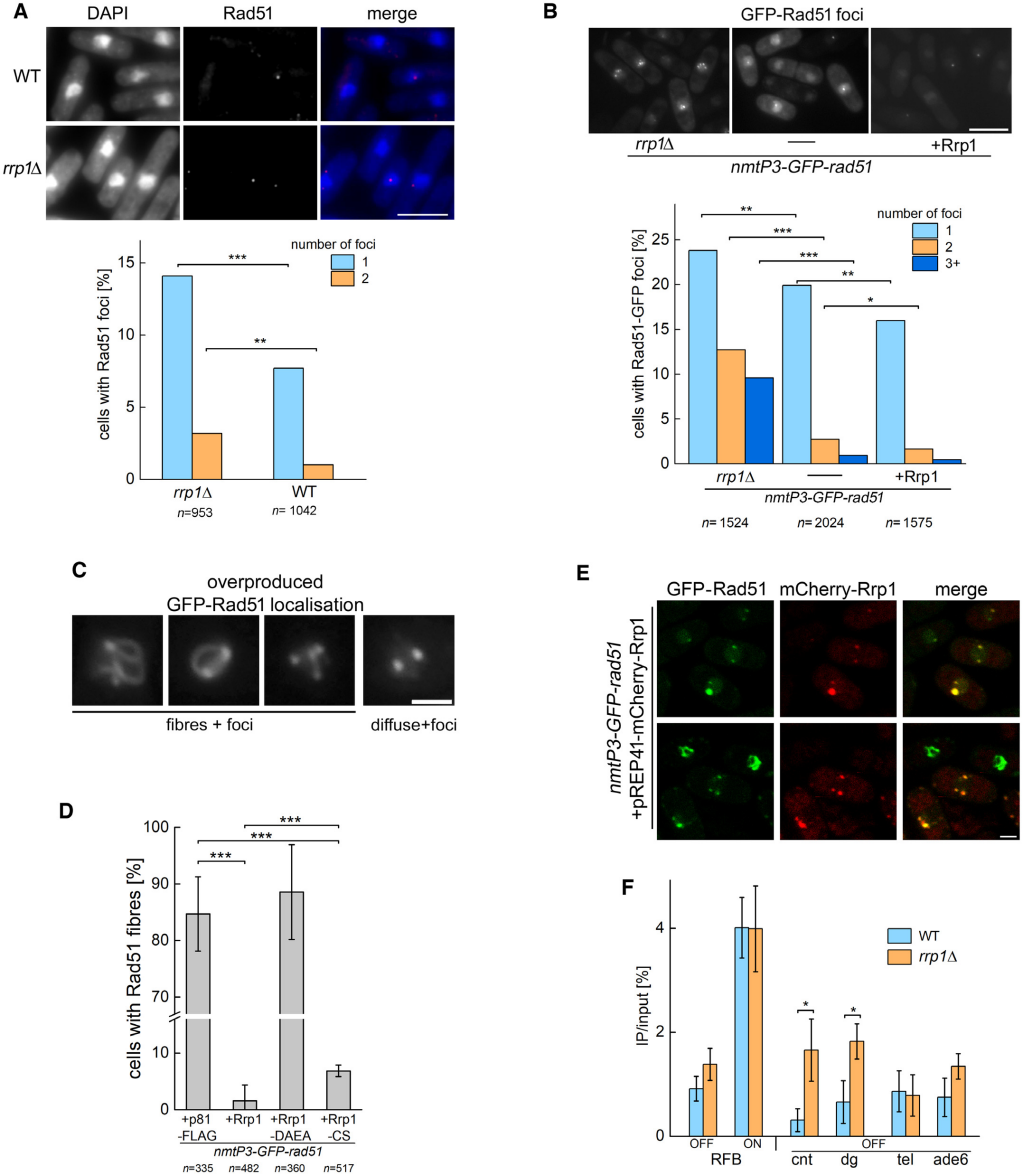
Rrp1 affects Rad51 localisation on chromatin. (**A**) *rrp1*^+^ deletion leads to an increase in the number of spontaneous native Rad51 foci. Rad51 spontaneous foci were detected by immunofluorescence microscopy with anti-Rad51 antibodies. DAPI stained nuclei are shown as a localisation control. n = total number of cells examined from two independent biological replicates. Scale bar represents 10 μm. (**B**) Under conditions of mild Rad51 overproduction, deletion of *rrp1*^+^ results in an increase in the number of cells with multiple GFP-Rad51 foci, and Rrp1 overproduction has the opposite effect. The *nmtP3-GFP-rad51* strain was cultured for 24 h on YES medium where the *nmtP3* promoter is mostly repressed. *n* = total number of cells examined from two independent experiments. Scale bar represents 10 μm. (**C, D**) Two patterns of GFP-Rad51 localisation are shown: long fibres connecting discrete foci, and diffuse staining with foci. Scale bar represents 2 μm. In cells overexpressing both *rrp1*^+^ and *GFP*-*rad51*^+^, long Rad51 fibres are no longer observed. This effect is dependent on a functional ATPase domain, while the RING domain may play a relatively minor role. *n* = total number of cells counted for three independent transformants of vector, *rrp1*^+^, *rrp1-DAEA or rrp1-CS* grown for 48 h in media lacking thiamine. The error bars represent the standard deviation about the mean values. The *Z*-test for two population proportions was used in all above assays to calculate the *Z*-statistic and corresponding *P*-values. (**E**) In cells overproducing both proteins, most GFP-Rad51 foci co-localise with mCherry-Rrp1 foci. Two panels with representative Rrp1 and Rad51 localisations are shown. Scale bar represents 2 μm. (F) Rrp1 influences Rad51 localisation specifically at the centromere. Analysis of Rad51 enrichment at indicated loci by ChIP-qPCR in wild-type and *rrp1*Δ strains, under conditions where a polar replication fork block was induced (–thi, ON) or repressed (+thi, OFF). Primers were located at an iducible replication fork barrier (RFB), telomeres (*tel*), outer repeat (*dg*) and core region (*cnt*) of centromeres and *ade6^+^*, a random euchromatin locus (*ade6*). Values are mean ± standard deviation from three independent biological replicates. Student's *t*-test was performed to calculate *P*-values. (*** *P* ≤ 0.001, ** 0.001 < *P* ≤ 0.01, * 0.01 < *P* ≤ 0.05)

It has been shown that endogenous Rad51 forms only few spontaneous foci in unchallenged cells (53–56). Consistently, we were able to detect such spontaneous Rad51 foci in approximately 10% of cells examined by immunofluorescence microscopy. This number increased twofold when *rrp1*^+^ was deleted (Figure 2A). Of note, cells exhibiting >1 focus were also more frequently observed in the absence of Rrp1.

To address modulation of Rad51 foci formation by Rrp1 in living cells, we took advantage of the *nmtP3-GFP-rad51* strain cultured on YES medium, where gene expression from the *nmtP3* promoter is very limited, and observed that 1–3 GFP-Rad51 foci were present in about 20% of cells (Figure 2B, top middle panel). Interestingly, deletion of *rrp1*^+^ not only led to an increase in the number of cells with one GFP-Rad51 focus but also resulted in a marked increase in the number of cells with multiple GFP-Rad51 foci (Figure 2B, top left panel). This correlates with exacerbation of the growth defect described above for *nmtP3-GFP-rad51 rrp1*Δ strain cultured on YES media (Figure 1A). The opposite was seen in *nmtP3-GFP-rad51* strain overexpressing *rrp1*^+^, where the number of cells with spontaneous GFP-Rad51 foci decreased (Figure 2B, top right panel). The effect was subtle but significant, especially given that *rrp1*^+^ expression is also very limited under these conditions.

Together, these results suggest that Rrp1 could be involved in the control of Rad51 binding to DNA. We were interested to determine which Rrp1 activity was necessary for this regulation but because of the strong growth defect observed in the *nmtP3-GFP-rad51 rrp1*Δ strain, accompanied by the rapid generation and subsequent proliferation of faster-growing suppressors, complementation analysis of its phenotype was not possible, so we again employed a synthetic dosage lethality approach.

It is known that when Rad51 is overproduced in human cells, it accumulates on chromatin, forming long fibres (35,57). We examined *GFP*-*rad51*^+^ overexpressing cells using fluorescence microscopy and observed that most of them contained extensive Rad51 fibres in their nuclei, often connecting several bright foci (Figure 2C). These structures virtually disappeared when Rrp1 was simultaneously overproduced, and GFP-Rad51 staining changed to diffuse with 1–3 foci (Figure 2C, D). Cells overproducing Rrp1-DAEA, an ATPase deficient mutant, contained GFP-Rad51 fibres in their nuclei (Figure 2D). This clearly demonstrates that the putative translocase activity of Rrp1 is critical in preventing excessive Rad51 accumulation on chromatin. Interestingly, even though overproduction of the Rrp1-CS mutant did supress the toxicity of *GFP-rad51*^+^ overexpression (Figure 1F), we were nevertheless able to detect a small, yet statistically significant, increase in the number of nuclei with Rad51 fibres in these cells (Figure 2D). This correlates with a significant increase in the number of cells with nuclear defects in the *nmtP3-GFP-rad51* strain overproducing Rrp1-CS as compared to wild-type Rrp1 (Figure 1F), and suggests that the putative Rrp1 ubiquitin ligase activity may also play a role in regulating Rad51 binding to DNA, although its effect is less prominent than that of the translocase activity.

When we simultaneously overproduced Rrp1-mCherry in the *nmtP3-GFP-rad51* strain, GFP-Rad51 fibres were visible only in cells lacking Rrp1 signal, and about 90% of GFP-Rad51 foci co-localised with Rrp1-mCherry foci (Figure 2E). We have previously shown that native Rrp1 is enriched at centromeres and >40% of the foci it forms when overproduced are perinuclear and co-localise with Swi6 foci (14). It therefore seemed possible that Rrp1 could interact with Rad51 bound to heterochromatin regions. Under standard growth conditions Rad51 binding may occur at sites of chromosomes where replication is perturbed and this can be more frequent at sites containing repetitive sequences, such as centromeres or telomeres, which are difficult to replicate. Indeed it has been shown in *S. pombe* that Rad51 binds to centromeres in S phase (58).

In order to determine if Rrp1 affects Rad51 binding to centromeres we examined by chromatin immunoprecipitation the effect of *rrp1*^+^ deletion on the ability of endogenous Rad51 to associate with outer repeat (*dg*) and core region (*cnt*) of centromeres. We added other selected loci such as telomeres (*tel*), as another heterochromatin genomic locus, *ade6*^+^ an euchromatin locus at which replication perturbation is not expected in unchallenged growth conditions (*ade*) and the inducible and engineered replication fork barrier (*RTS1*-RFB) that allows a single replisome to be blocked in a polar manner on chromosome III (27). The activity of the RFB is regulated by the *RTS1*-bound Rtf1 protein which expression is repressed in the presence of thiamine (RFB OFF) and induced in the absence of thiamine (RFB ON).

Consistent with previous reports (59,60), Rad51 was enriched at the active RFB (RFB ON) as compared to RFB OFF condition (Figure 2F). We observed similar level of enrichment in WT and *rrp1Δ* strains, indicating that Rrp1 does not regulate Rad51 binding at this site-specific fork arrest. In contrast, we observed that Rad51 was significantly more enriched at both outer repeat (*dg*) and core region (*cnt*) of centromeres in *rrp1Δ* than in *WT* cells (*P-*values 0.036 and 0.04 for *dg* and *cnt*, respectively) whereas no higher enrichment was observed at telomeres and *ade6*^+^ locus (*P-*values 0.85 and 0.13 for *tel* and *ade6*, respectively) (Figure 2F).

This result supports the notion that Rrp1 regulates Rad51 bound to specific sites or DNA structures, such as those present within centromeric regions. This agrees with the previously proposed role for Rrp1 in maintaining centromere stability (14), although further studies are needed to examine how these Rrp1 activities are connected.

### The role of Rrp1 in regulating Rad51-induced toxicity is independent from Rrp2

Overproduction of Rrp2 in a *rad51^+^* overexpressing strain was unable to suppress the growth defect (Figure 1C, D), viability loss (Supplementary Figure S1A, C) and the chromosome segregation defect (Supplementary Figure S1F), and did not prevent the accumulation of Rad51 fibres on chromatin (Supplementary Figure S1G). Moreover, rescue of the GFP-*rad51^+^* overexpression-induced growth defect by overproduction of Rrp1 was not affected by the presence of Rrp2 (Supplementary Figure S1H) or the recombination auxiliary factor complex Swi5-Sfr1 (Supplementary Figure S1I). This suggests that the observed role of Rrp1 in regulating Rad51 is distinct from the previously described mutually dependent role of Rrp1 and Rrp2 in the Swi5-Sfr1 sub-pathway of HR (12,13).

### Purified Rrp1 binds to DNA and has a DNA-dependent ATPase activity

In order to gain mechanistic insight into the function of Rrp1, recombinant Rrp1-FLAG was purified to near-homogeneity following overexpression in *Escherichia coli* (Supplementary Figure S2A).

Since Rrp1 was predicted to have ATPase activity (61), we first examined if the purified protein could indeed hydrolyse ATP. In the absence of DNA, very low ATPase activity was detected. However, robust ATP hydrolysis was observed in the presence of dsDNA and a lower ATPase activity was seen in the presence of short oligo ssDNA (Supplementary Figure S2B). These results indicate that Rrp1 has an ATPase activity preferentially stimulated by dsDNA. In order to confirm that the observed ATPase activity is not derived from contaminating protein(s) in the Rrp1 protein preparation, we measured the ATPase activity and protein concentration in the peak fractions from the final purification step. The dsDNA-dependent and -independent ATPase activities corresponded with protein concentration signal for Rrp1, indicating that the observed ATPase activities are intrinsic properties of the Rrp1 protein (Supplementary Figure S2C).

The observed dependency on DNA for ATP hydrolysis suggested that Rrp1 is capable of binding DNA. This was tested by electrophoretic mobility-shift assays (EMSA). At concentrations as low as 0.05 μM (Rrp1: nucleotide ratio of 1:100), all circular ssDNA (cssDNA) was shifted in an ATP-independent manner by Rrp1, and this shift was enhanced at higher concentrations of protein (Supplementary Figure S2D). A lesser shift was observed with linearized dsDNA (ldsDNA), with some unbound DNA remaining even at 0.30 μM Rrp1 (Rrp1: base pair ratio of 1:8.33), although ATP was also dispensable for this binding (Supplementary Figure S2E). Some signal was observed in the wells, particularly for dsDNA, suggesting that Rrp1 may form aggregates consisting of protein-DNA networks that are too large to enter the gel. Rrp1 binding to dsDNA lacking free DNA ends (covalently closed circular DNA, cccDNA) was comparable to cssDNA, with the exception that no shift was observed at 0.05 μM of Rrp1 (Supplementary Figure S2F). Taken together, these results indicate that Rrp1 binds both ssDNA and dsDNA in an ATP-independent manner, with slightly higher affinity for ssDNA.

### Rrp1 physically interacts with Rad51

**Figure 3.**
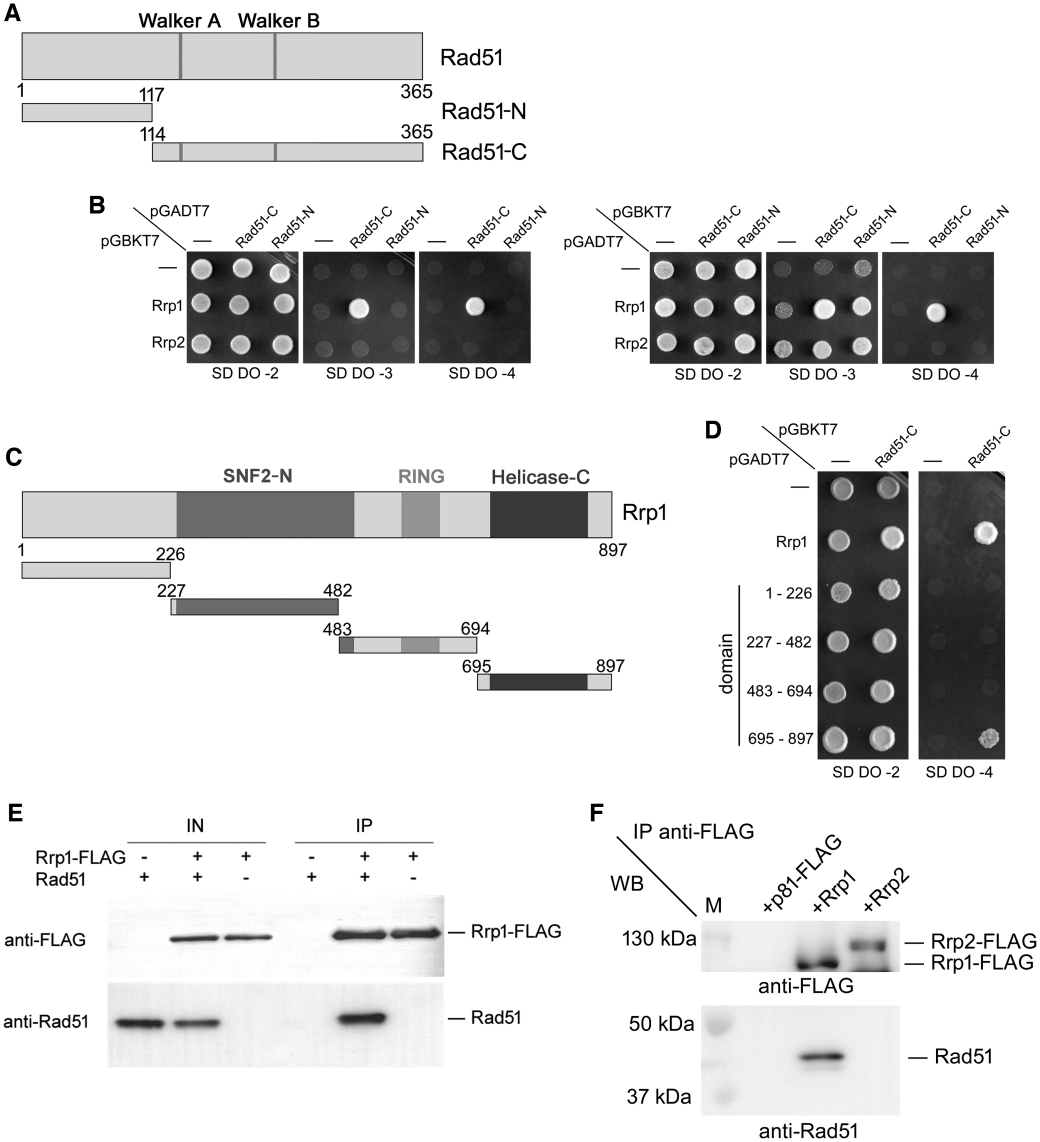
Rrp1 directly interacts with Rad51. Analysis of the Rrp1 interaction with Rad51 by yeast two-hybrid analysis. (**A**) Two Rad51 constructs were used: Rad51-N (N-terminal, residues 1–117) and Rad51-C (C-terminal, residues 114–365 containing the Walker A and B motifs). (**B**) Rad51-C (pGBKT7 and pGADT7 plasmid) is involved in the interaction with Rrp1 (self-activation is observed in the transformant with pGADT7-Rrp1 and empty pGBKT7 vector, right panels, suggestive of Rrp1 DNA binding activity). Transformants were selected on synthetic dextrose drop-out medium without Leu and Trp (SD DO-2), then plated on low stringency medium without Leu, Trp and His (SD DO-3) and high stringency medium without Leu, Trp, His and Ade (SD DO-4). (**C**) Schematic representation of a series of four truncated forms of Rrp1 used to map the site of interaction with Rad51. (**D**) The C-terminal fragment of Rrp1 (residues 695–897) containing the C-helicase domain was found to interact with Rad51, determined as described for (B). (**E**) Rad51 immunoprecipitated with Rrp1-FLAG *in vitro*. Purified Rad51 and purified Rrp1-FLAG were mixed and incubated with anti-FLAG M2 agarose. Proteins were eluted from the beads with 3xFLAG peptide and separated by SDS-PAGE, then analysed by Western with anti-FLAG antibody and anti-Rad51 antiserum. -, protein was omitted and the equivalent volume of protein storage buffer was added instead. +, protein was included. The experiment was repeated twice. (**F**) Rad51 interacts with Rrp1-FLAG, but not with Rrp2-FLAG, *in vivo*. Protein extracts prepared from cells overexpressing either *rrp1-FLAG* (three independent transformants were examined) or *rrp2-FLAG*, or transformed with empty vector as a control (pREP81-FLAG plasmid), were incubated with anti-FLAG M2 agarose. Proteins were then eluted with 3xFLAG peptide, separated by SDS-PAGE, and analysed by Western with anti-FLAG antibody and anti-Rad51 antiserum.

The genetic interactions observed between Rad51 and Rrp1 raised the possibility that the two proteins interact physically. To investigate this possibility, we first employed the yeast two-hybrid system (Y2H). For Rad51, two constructs were used: a short N-terminal fragment (Rad51-N), and a long C-terminal fragment containing the Walker A and B motifs (Rad51-C) (Figure 3A). We observed a robust growth of transformants containing genes for Rrp1 and Rad51-C on high stringency SD DO-4 plates, indicating that the site of putative Rrp1 binding lies within the Rad51 region containing Walker A and B motifs (Figure 3B). In order to map the corresponding region within Rrp1 that is responsible for Rad51 binding, we created a series of four truncated forms of Rrp1 (Figure 3C) and repeated the Y2H assay with Rad51-C. These experiments revealed that the fragment of Rrp1 containing its C-terminal helicase domain was sufficient for the interaction with Rad51 (Figure 3D). In agreement with our genetic data, no interaction was observed between Rad51 and Rrp2 by Y2H (Figure 3B).

To validate these Y2H results and rule-out the possibility that the observed Rrp1-Rad51 interaction involved an intermediary molecule, purified Rad51 and purified Rrp1-FLAG were mixed together and subjected to immunoprecipitation with anti-FLAG M2 agarose. Rad51 was seen to co-immunoprecipitate with Rrp1-FLAG (Figure 3E), thus confirming the existence of a direct interaction between these two proteins. Furthermore, by immunoprecipitating endogenously expressed Rad51 with overproduced Rrp1-FLAG from native protein extracts, we were able to demonstrate that the Rad51-Rrp1 complex is formed *in vivo* in *S. pombe* cells (Figure 3F). We did not see such complex formation between Rad51 and Rrp2-FLAG.

### Rrp1 dissociates Rad51 from dsDNA *in vitro*

**Figure 4.**
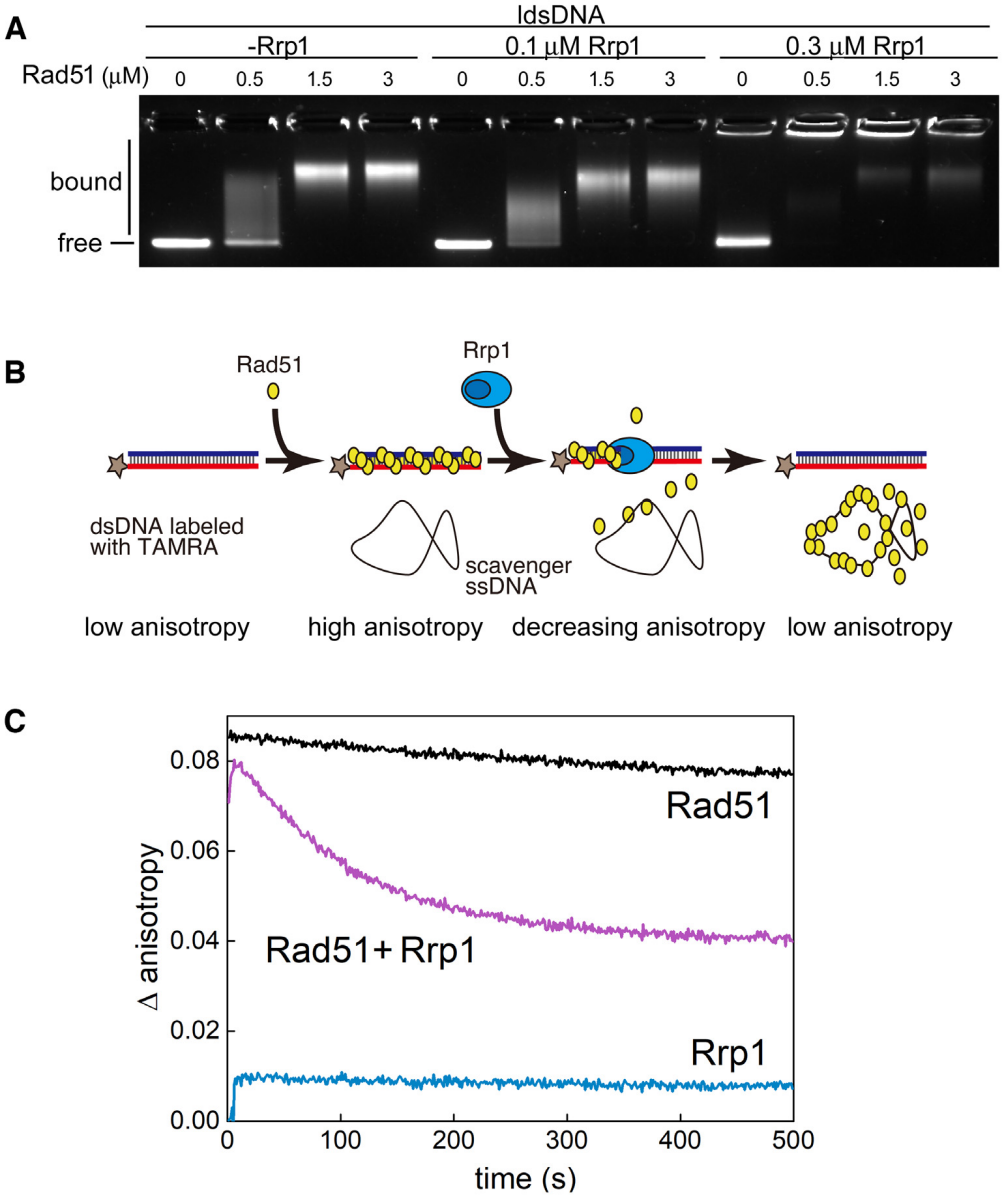
Rrp1 can disassemble Rad51-dsDNA complexes. (**A**) Rrp1 outcompetes Rad51 for binding to dsDNA as demonstrated by electrophoretic mobility shift assay (EMSA). Increasing amounts of Rad51 were pre-incubated with linear double-stranded DNA (ldsDNA) before addition of the indicated concentration of Rrp1. Mixtures were resolved on an agarose gel and stained with SYBR-gold. (**B**) Diagram of the fluorescence anisotropy assay used to measure in real-time the dissociation of Rad51 from fluorescently labelled dsDNA. Rad51 (6 μM) was incubated with a dsDNA oligonucleotide (3 μM base pair concentration) labelled with the TAMRA fluorophore. Unlabelled heterologous scavenger ssDNA was then added, followed by a sub-stoichiometric amount of Rrp1 (0.25 μM) or the equivalent volume of protein storage buffer, and fluorescence anisotropy was monitored. (**C**) Time course of Rad51 disassembly from dsDNA following the addition of Rrp1 was performed for two independent Rrp1 preparations. A representative result is shown, with the comparative decline in anisotropy (Δ anisotropy) observed in the three independent reactions containing Rrp1, indicating that Rad51–dsDNA complexes are disassembled.

SWI2/SNF2-related translocases have been proposed to remove Rad51 from dsDNA in heteroduplex DNA and dead-end non-productive complexes, both in yeast and human cells (35,39). The existence of a physical interaction between Rrp1 and Rad51, combined with the ability of Rrp1 to suppress erroneous association of Rad51 with chromatin (Figures 2 and 3), prompted us to examine whether Rrp1 can directly counteract Rad51 binding to linearized dsDNA. To test this, we exploited the fact that the binding of purified Rrp1 to dsDNA results in a distinctive EMSA pattern where most of the dsDNA signal is retained in the well (Supplementary Figure S2E). This pattern is easily distinguishable from the binding of Rad51 to DNA, which produces a smear at low concentrations of protein (0.5 μM) and a discrete band at higher concentrations (1.5 or 3 μM; Figure 4A). dsDNA was first coated with different concentrations of Rad51 and then challenged with sub-stoichiometric amounts of Rrp1. Protein-DNA complexes were then resolved by gel agarose electrophoresis. Compared to the condition where Rrp1 was omitted, the bands for Rad51-bound DNA became fainter when 0.1 μM of Rrp1 was included. Moreover, the inclusion of 0.3 μM of Rrp1 led to a drastic decline in the signal of Rad51-dsDNA bands, even when the dsDNA was precoated with five-fold more Rad51 molecules, and signal in the well became apparent. These results suggest that sub-stoichiometric amounts of Rrp1 effectively outcompete Rad51 for binding to dsDNA. Since Rrp1 can bind to both dsDNA (Supplementary Figure S2E, F) and Rad51 (Figure 3E), an alternative explanation for these results is that, rather than displace Rad51 from dsDNA, Rrp1 binds to Rad51-dsDNA complexes, leading to the formation of higher-order complexes that are unable to enter the gel.

It has been shown before that Rad51 protein strongly binds to dsDNA and its turnover is low both in yeast and humans (62,63). Nevertheless, the addition of scavenger DNA helps to visualise dissociation of Rad51 molecules (64). Thus, in order to more directly examine if Rrp1 could remove Rad51 from dsDNA, we analysed dissociation of Rad51 from fluorescently labeled dsDNA by measuring fluorescence anisotropy in real-time in the presence of a vast excess of scavenger ssDNA (Figure 4B). In this assay, Rad51 was first bound to fluorescently-labeled dsDNA to form a filament. This induced a decrease in the mobility of dsDNA, observed as an increase in fluorescence anisotropy (Δ anisotropy value of approximately 0.1). Even after the addition of scavenger ssDNA, the Δ anisotropy values decreased only slightly over 500s (black line in Figure 4C), indicating that the Rad51–dsDNA filament is relatively stable, consistent with reports described above. Upon Rrp1 addition to the reaction mixture containing the Rad51-dsDNA filament and scavenger ssDNA, the Δ anisotropy values decreased rapidly, indicating that Rad51 was being cleared from dsDNA (purple line in Figure 4C). A fit with $ \propto$exp(–*k*_off_ ·*t*) function gave a good approximation to our experimental data and yielded *k*_off_ rate, corresponding to dissociation rate, for Rad51, *k*_off_ = 1.63 × 10^–3^ s^–1^, (fit quality, *R*^2^ = 0.948) and for the removal of Rad51 by Rrp1, *k*_off_ = (9.46 ± 0.53) × 10^–3^ s^–1^, (fit quality, *R*^2^ > 0.994). This allowed us to estimate that in our experimental setup Rad51 dissociates from dsDNA almost six-fold faster in the presence of Rrp1. While further extensive, in-depth analysis is required to determine the influence of Rrp1 on Rad51 dissociation kinetics, our data directly demonstrate that Rrp1 is involved in the removal Rad51 from dsDNA.

A control experiment showed that when the same amount of Rrp1 was added to the dsDNA alone in the absence of Rad51, it elicited a slight increase in anisotropy (blue line in Figure 4C), thus ruling out the possibility that Rrp1 modifies the dsDNA in some way that reduces anisotropy.

We also examined the effect of Rrp1 on Rad51–ssDNA complexes. Although a slight decrease in the intensity of Rad51-ssDNA bands was observed by EMSA (Supplementary Figure S3A), this was negligible when compared with the effect of Rrp1 on Rad51–dsDNA complexes (Figure 4A). Consistently, relative anisotropy increased upon addition of Rrp1 to Rad51–ssDNA complexes in a concentration-dependent manner (Supplementary Figure S3B), suggesting that rather than dissembling Rad51–ssDNA complexes, Rrp1 was binding to them. This binding to Rad51–ssDNA filaments may lead to modulation of their activity, but further studies are needed to conclusively test this hypothesis.

### Rrp1 has an E3 ubiquitin ligase activity with Rad51 as a substrate

In addition to an ATPase domain, Rrp1 also has a Zinc finger RING-type domain characteristic of E3 ubiquitin ligases (61), and belongs, together with its *S. cerevisiae* orthologue Uls1, to the RING-domain-containing Rad5/16-like group of SWI2/SNF2 translocases, distinct from the RAD54 family (65,66).

**Figure 5.**
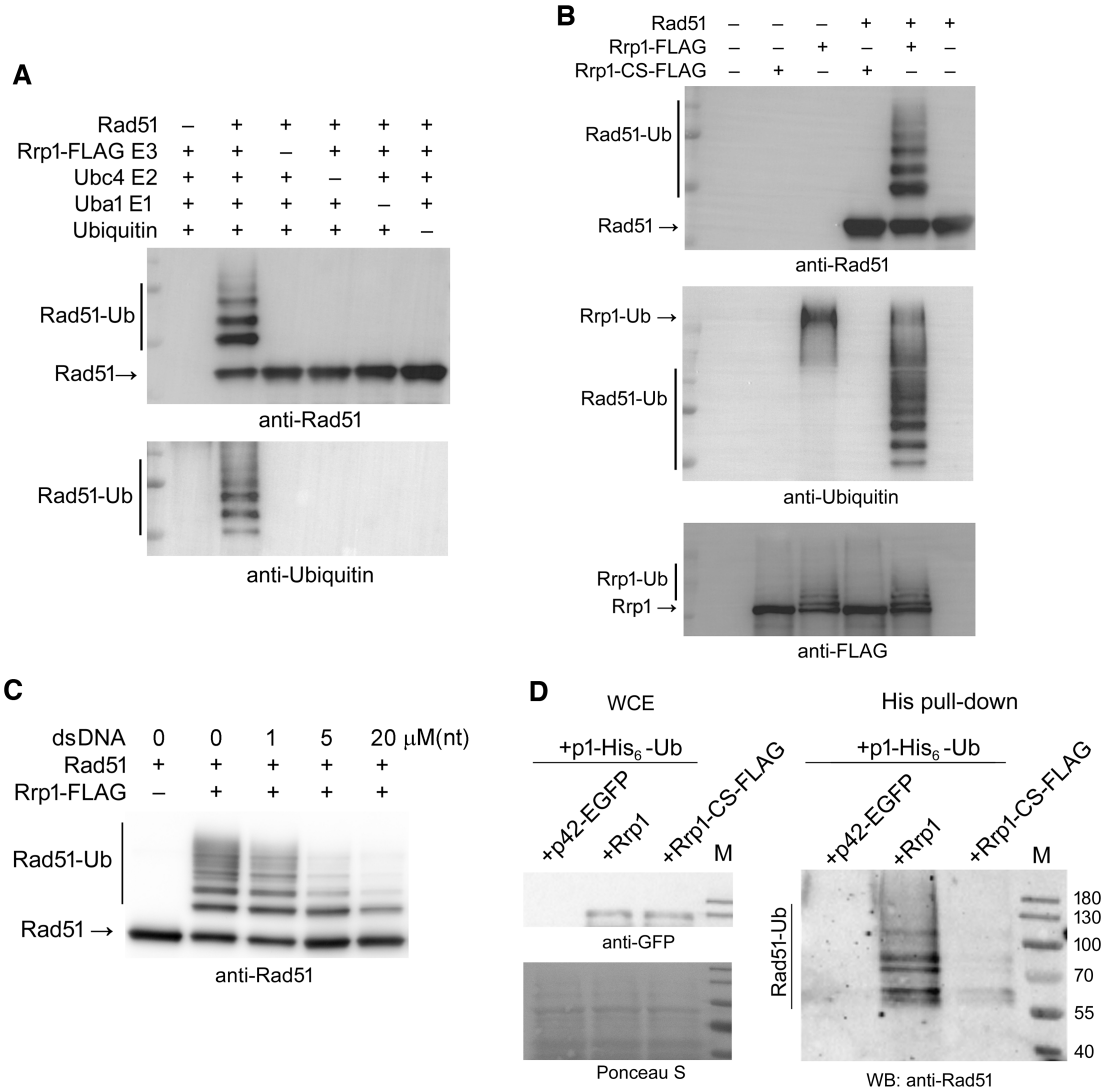
Rrp1 is an E3 ubiquitin ligase with Rad51 as a substrate. (**A**) Rrp1 ubiquitylates Rad51 *in vitro*. The indicated reaction components were included (+) or omitted (-) for *in vitro* ubiquitylation assays. After the reaction, the reaction mixture was analysed by western blotting with anti-Rad51 antiserum and anti-Ubiquitin antibodies, and multiple bands indicative of Rad51 ubiquitylation (Rad51-Ub) are shown. (**B**) Rrp1 RING domain is critical for Rad51 ubiquitylation. *In vitro* ubiquitylation assay containing all components as in (A) with Rrp1-FLAG or Rrp1-CS-FLAG as the E3 ligase. The reaction mixture was analysed by western blotting with anti-Rad51 antiserum and anti-Ubiquitin antibodies. Additionally, reaction products were analysed with anti-FLAG antibodies (lowest panel), revealing auto-ubiquitylation of Rrp1 (Rrp1-Ub). (**C**) The presence of DNA limits ubiquitylation of Rad51 by Rrp1. *In vitro* ubiquitylation assay containing all components as in (A) with pre-formed Rad51-dsDNA complexes obtained by incubation of 1 μM Rad51 with indicated concentrations of dsDNA. The reaction mixture was analysed by western blotting with anti-Rad51 antiserum. (**D**) Rrp1 overproduction leads to the accumulation of ubiquitylated forms of Rad51 *in vivo*. The wild-type strain (WT) was co-transformed with a pREP1-based plasmid encoding hexahistidine-tagged Ubiquitin (+p1-His_6_-Ub) together with pREP42 based plasmid (p42-EGFP) harbouring genes for Rrp1 or Rrp1-CS-FLAG. After 24 h growth under expression inducing conditions, ubiquitylated proteins were isolated by His pull-down and detected by western blot with Rad51 antiserum. Experiment was repeated twice for independent sets of transformants. Rrp1 and Rrp1-CS protein levels were comparable, as seen in whole cell extracts (WCE) probed with anti-GFP antibodies.

We therefore hypothesized that Rrp1 may have an E3 ligase activity with Rad51 as a substrate. In order to directly test this possibility, *in vitro* Rad51 ubiquitylation assays were performed using ubiquitin, Uba1 (E1) and Ubc4 (E2) enzymes purified from *E. coli*, and purified Rrp1-FLAG as the sole E3 ubiquitin ligase enzyme. Reaction products were separated by SDS-PAGE and subjected to western blot analysis. Multiple high molecular weight protein bands were observed only when all assay components were included in the reaction (Figure 5A). Strikingly, a similar banding pattern was detected with both anti-Rad51 and anti-Ubiquitin antibodies, indicating that these bands represent ubiquitylated forms of Rad51. Consistent with this notion, the unmodified Rad51 band decreased in intensity only in the assay with all reaction components (Figure 5A). To further validate these findings, we repeated this ubiquitylation assay with either wild-type Rrp1 protein or the Rrp1-CS variant, where the Rrp1 RING domain was inactivated, as the sole E3 ligase. When Rrp1-CS was employed, the characteristic protein ladder was not formed (Figure 5B), indicating that Rrp1 E3 ubiquitin ligase activity was responsible for Rad51 ubiquitylation.

Interestingly, when the membrane was probed with an anti-FLAG antibody to detect Rrp1 protein, several high molecular weight protein bands greater in mass than Rrp1 were observed in reactions containing wild-type Rrp1 but not Rrp1-CS (Figure 5B, bottom panel), indicating that Rrp1 is capable of auto-ubiquitylation. Consistent with this notion, the intensity of the unmodified Rrp1 band was decreased in these reactions.

When the Rad51 ubiquitylation assay was performed with Rad51 preassembled on dsDNA, the intensity of bands corresponding to ubiquitylated Rad51 decreased in a dose dependent manner with increasing concentrations of DNA (Figure 5C). This was especially evident for poly-ubiquitylated Rad51 species, and demonstrates that Rad51 ubiquitylation by Rrp1 is inhibited by dsDNA. The precise mechanism however, whereby dsDNA inhibits this ubiquitylation, remains to be elucidated.

In order to determine if Rrp1-dependent ubiquitylation of endogenous Rad51 can be detected *in vivo*, we prepared protein extracts from cells expressing His_6_-tagged Ubiquitin from a strong *nmt* promoter (*nmt1*) and overproducing Rrp1. Precipitates from the His pull-down fractions were analysed by Western blot and probed with anti-Rad51 antibody. We observed an accumulation of high molecular weight Rad51 species, and these were substantially less abundant in cells overproducing the Rrp1-CS mutant protein, or carrying an empty vector (Figure 5D). This bolsters our *in vitro* observations (Figure 5A, B) that Rrp1 has the ability to ubiquitylate Rad51 in a manner dependent on its functional RING domain.

Polyubiquitylation is a well-established signal for protein degradation (67). We did not observe by Western blot any significant changes in native Rad51 protein levels in total extract fractions upon *rrp1*^+^ deletion or overexpression (Supplementary Figure S4A, upper-right graph). However, after prolonged overexpression of *rrp1*-CS, Rad51 accumulation could be detected (Supplementary Figure S4A bottom-left graph). Rad51 levels increased both in nucleoplasm and chromatin fractions obtained from cells overexpressing *rrp1*-CS, (Supplementary Figure S4B), suggesting that Rrp1 may have a role in Rad51 turnover that is dependent on its ubiquitin ligase activity.

This result is somewhat surprising since overproduction of wild-type Rrp1 does not lead to a reduction in Rad51 levels that is detectable by Western blotting. We propose that under standard growth conditions Rrp1 modulates the activity of only a small fraction of Rad51 molecules, as was shown by Rad51-ChIP (Figure 2F), meaning that global Rad51 turnover is virtually unaffected by Rrp1 overproduction. Only when degradation of this subset of Rad51 molecules, presumably removed from DNA by Rrp1, is defective, as in the case of prolonged overexpression of *rrp1*-CS, limited Rad51 accumulation can be detected by Western blotting.

In this work we have demonstrated that high levels of Rad51 are toxic to *S. pombe* cells and, as discussed above, *rrp1*-CS overexpression results in the increase in Rad51 levels. Yet, as shown previously (14) *rrp1*-CS overexpression does not lead to a growth defect. We reason that other ubiquitin ligases might be involved in removing excess Rad51 from cells overproducing Rrp1-CS mutant, so Rad51 levels on DNA do not rise above the threshold that would lead to growth inhibition. Indeed, in the *fbh1*Δ strain, which is devoid of a component of an E3 complex that can ubiquitylate Rad51 (44), overexpression of *rrp1*-CS is more toxic than overexpression of wild-type *rrp1*+ (Supplementary Figure S4C).

We thus identify Rrp1 as an ATPase and translocase that can remove Rad51 from dsDNA, and as a ubiquitin ligase with Rad51 as its substrate. We propose that these Rrp1 activities are important for the regulation of Rad51 function.

## DISCUSSION

Previous studies have shown that the presence of the RAD54 family of SWI2/SNF2 DNA translocases, RAD54L and RAD54B in humans (35), and Rdh54 and Rad54 in *S. cerevisiae* (39), is necessary to counteract the genotoxic effects of Rad51 overproduction. Another budding yeast protein Uls1, has also been shown to participate in modulating Rad51 activity. *S. pombe* Rrp1 and Rrp2 are Uls1 orthologues and all three proteins belong to a unique Rad5/16-like group of SWI2/SNF2 DNA translocases, that contain both an ATPase domain and a Zinc finger RING-type domain characteristic of E3 ubiquitin ligases (65,66).

In this work, we demonstrated that toxicity of *rad51*^+^ overexpression was increased in a *rrp1*Δ but not in a *rrp2*Δ strain. This implied that, in *S. pombe*, Rrp1 has a prominent function, independent from Rrp2, in counteracting the toxicity of Rad51 overproduction. This is in contrast to the requirement for Uls1 in *S. cerevisiae*, which only becomes apparent in the absence of both Rdh54 and Rad54 (39). We thus examined the contributions of putative translocase and ubiquitin ligase activities of Rrp1 to its interaction with Rad51.

We found that counteracting the cellular toxicity of Rad51 overexpression requires the ATPase but not the RING domain. Similarly, the deleterious consequences of Rad51 overexpression on chromosome segregation are rescued via the ATPase domain of Rrp1, suggesting that Rrp1 modulates Rad51 through its translocase activity. Interestingly, however, we found that the Rrp1-CS mutant, with an inactivated RING domain, was slightly less proficient in counteracting inappropriate Rad51 accumulation on chromatin and the appearance of aberrant DNA segregation events. This raised an interesting possibility, not examined for its orthologue Uls1, that Rrp1 ubiquitin ligase activity may play a role in Rad51 regulation, even though it was apparently not crucial for suppression of the Rad51 overproduction-induced growth defect.

Importantly, Rrp1 is also involved in regulating Rad51 when the latter is not overproduced. We found that the number of spontaneous Rad51 foci under endogenous expression conditions increased in cells lacking Rrp1. Moreover, Rad51-ChIP demonstrated that Rrp1 has no global effect on Rad51 binding, and does not influence its levels even at blocked replication forks, where Rad51 is significantly enriched. Instead, Rrp1 specifically affects the ability of endogenous Rad51 to associate with centromeres. This suggests that the physiological role of Rrp1 may be to regulate a subpopulation of Rad51 molecules bound to specific sites or DNA structures, such as those present within centromeric regions. It has been previously shown that *S. pombe* Rad51 localises to centromeres (58), and together with another SWI2/SNF2 translocase, Rad54, promotes recombination between centromere repeats that is important for chromosome stability (68). This indicates that two SWI2/SNF2 translocases, Rad54 and Rrp1, may participate in Rad51 regulation that is important for the maintenance of centromere function.

Purification of Rrp1 allowed us to obtain mechanistic insight into its function and biochemical activities. Purified Rrp1 binds to both ssDNA and dsDNA independently of ATP, and has a DNA-dependent ATPase activity. Importantly, we demonstrated that Rrp1 and Rad51 interact both *in vitro* and *in vivo*, supporting our conclusion that Rad51 may be the direct target of Rrp1 activity. Indeed, using an *in vitro* fluorescence anisotropy assay, we showed that Rrp1 can efficiently dissociate Rad51 from dsDNA, establishing Rrp1 as a translocase that counteracts Rad51 binding to chromatin to limit its genotoxicity. Our data thus indicate that the Rad5/16-like group of SWI2/SNF2 translocases also participates in limiting Rad51-mediated toxicity, as shown for RAD54-like DNA translocases (35).

Our work suggests that the RING domain of Rrp1 may also be involved in Rad51 regulation. We provide direct evidence that Rad51 undergoes polyubiquitylation that is dependent on the RING domain of Rrp1, *in vivo* and *in vitro*. This indicates that Rrp1 has an E3 ubiquitin ligase activity and Rad51 is one of its substrates.

Because the RING domain is mostly dispensable to counteract the toxicity of Rad51 overexpression, we propose that Rad51 ubiquitylation by Rrp1 is not an absolute prerequisite for Rad51 removal from DNA by Rrp1’s translocase activity. Nevertheless, examination of *rad51*^+^-overexpressing cells simultaneously overproducing Rrp1-CS or wild-type Rrp1 revealed that the number of cells with Rad51 fibres and segregation defects was increased in the former, implying that inactivation of the RING domain does affect the ability of Rrp1 to prevent Rad51 association with DNA.

Rad51 ubiquitylation has previously been shown to compromise its ability to bind DNA (43,45) and it has been proposed that the FBH1 translocase displaces RAD51 from ssDNA and ubiquitylates it to prevent its reloading (45). Since the efficiency of Rrp1-mediated ubiquitylation of Rad51 preassembled on dsDNA was markedly decreased, we speculate that a similar two-step model could be applicable for Rrp1: Rrp1 translocase could displace Rad51 from dsDNA and ubiquitylate it to prevent its reloading. However, since we do not know how DNA inhibits Rad51 ubiquitylation, more studies are needed to understand how the Rrp1 ATPase/translocase and ubiquitin ligase activities cooperate in counteracting excessive Rad51 binding to chromatin.

Rad51 polyubiquitylation by RFWD3 not only inhibits its DNA binding but also leads to its proteasomal degradation (43). In accord with our assumption that Rrp1, which is an extremely low abundance protein, interacts only with a small fraction of Rad51 molecules and does not affect global Rad51 turnover, we did not detect any significant changes in native Rad51 protein levels upon *rrp1*^+^ deletion or overexpression. However, Rad51 levels increased in cells overexpressing *rrp1*-CS, suggesting that Rrp1 ubiquitin ligase activity may have a role in promoting the proteolytic degradation of a specific subset of Rad51 molecules.

Members of the Rad5/16-like group of SWI2/SNF2 translocases, such as budding yeast Rad5 and human SHPRH and HLTF, have been shown thus far to ubiquitylate PCNA (69). Recently, SHPRH has been identified as a nucleosome E3-ubiquitin-ligase (70), and we have demonstrated that Rrp1 is involved in modulation of nucleosome dynamics important for centromere, and thus genome, stability (14). Our present work, describing the Rrp1-Rad51 interaction, which involves ATPase/translocase and ubiquitin ligase activities of Rrp1, extends the possible range of functions performed by this class of SNF2 enzymes and may contribute to a better understanding of their role in modulating specific activities at perturbed replication forks and at regions of the genome that are difficult to replicate, such as centromeres.

Rad51 is overproduced in several types of human cancers (71,72) and multiple cancer cell lines (73,74), and contributes to their increased survival after DSB induction. Increased levels of Rad51 may compensate for deficiencies in other DNA repair pathways in cancer cells and are often associated with poor patient survival prognosis (75,76). Since the role of ubiquitylation has not been addressed in previous studies on Rad51 dysregulation by Swi2/Snf2-related translocases (35,39), our work may have implications for human health.

## DATA AVAILABILITY

Source data as well as strains and plasmids are available upon request.

## Supplementary Material

gkab511_Supplemental_File

## Notes

Present address: Bilge Argunhan, Section of Structural Biology, Faculty of Medicine, Imperial College London, UK.

Present address: Yumiko Kurokawa and Yasuto Murayama, Center for Frontier Research, National Institute of Genetics, Japan.
