# Supplementary material for: Rrp1 translocase and ubiquitin ligase activities restrict the genome destabilising effects of Rad51 in fission yeast
Source: Nucleic Acids Res. 2021 Jun 22;49(12):6832–48. doi: 10.1093/nar/gkab511 (PMC8266636; doi:10.1093/nar/gkab511)
Supplement: gkab511_Supplemental_File [file gkab511_supplemental_file.pdf]

Supplementary material for:

Rrp1 translocase and ubiquitin ligase activities restrict the genome destabilising effects of Rad51 in fission yeast

Jakub Muraszko, Karol Kramarz, Bilge Argunhan, Kentaro Ito, Gabriela Baranowska, Yumiko Kurokawa, Yasuto Murayama, Hideo Tsubouchi, Sarah Lambert, Hiroshi Iwasaki and Dorota Dziadkowiec

Table S1 Strains used in this study

Strain	Genotype	Reference
YA254 (WT)	ura4-D18, leu1-32, his3-D1, arg3-D1, h90	a
rrp1Δ	rrp1D::natMX, his3-D1, leu1-32, ura4-D18, arg3-D1, h90	lab stock
rrp2Δ	rrp2D::natMX6, his3-D1, leu1-32, ura4-D18, arg3-D1, h90	lab stock
SP282 *nmtP3-GFP-rad51*	kan-nmtP3-GFP-Rad51, leu1-32, ura4-D18, h-	b
nmtP3-GFP-rad51	kan-nmtP3-GFP-Rad51, his3-D1, leu1-32, ura4-D18, arg3-D1, h-	c
nmtP3-GFP-rad51 rrp1Δ	kan-nmtP3-GFP-Rad51, rrp1D::kanMX6, his3-D1, leu1-32, ura4-D18, arg3-D1, h-	c
nmtP3-GFP-rad51 rrp2Δ	kan-nmtP3-GFP-Rad51, rrp2D::kanMX6, his3-D1, leu1-32, ura4-D18, arg3-D1, h-	c
nmtP3-GFP-rad51 p42-rrp2-HA	kan-nmtP3-GFP-Rad51, ars1(MluI)::pREP42-HA-Rrp2, ura4+, his3-D1, leu1-32, ura4-D18, arg3-D1, h-	c
nmtP3-GFP-rad51 sfr1Δ	kan-nmtP3-GFP-Rad51, sfr1D::arg3, arg3-D4, ura4-D18, leu1-32, h-	c
nmtP3-GFP-rad51 swi5Δ	kan-nmtP3-GFP-Rad51, swi5D::his3+, ura4-D18, leu1-32, his3-D1, arg3-D1, h-	c
KK396 RTS1-RFB	nmt41:Rtf1:sup35, ura4-sd20-RTS1, ade6-704, leu1-32, h+	d
KK400 *rrp1Δ* RTS1-RFB	rrp1D::HygroMX, nmt41:Rtf1:sup35, ura4-sd20-RTS1, ade6-704, leu1-32, h-	d
SL857 *fbh1Δ*	fbh1::natMX6, ade6-704, leu1-32, ura4-D18, h+	d
S. cerevisiae AH109	MATa, trp1-901, leu2-3, 112, ura3-52, his3-200, gal4D, gal80D, LYS2::GAL1UAS-GAL1TATA HIS3, GAL2UASGAL2TATA-ADE2, URA3::MEL1UAS-MEL1TATA-lacZ	CLONTECH
E. coli Rosetta(DE3)	*F- ompT hsdSB(rB- mB-) gal dcm (DE3) pRARE (CamR)*	Novagen

a - Akamatsu,Y., Dziadkowiec,D., Ikeguchi,M., Shinagawa,H. and Iwasaki,H. (2003) Proc. Natl. Acad. Sci. U. S. A., 100, 15770–5.
b - Hiroshi Iwasaki laboratory
c - this work
d - Sarah Lambert laboratory

Table S2 Plasmids used in this study

Plasmid	Reference
pREP41-mCherry	a
pREP41-mCherry -Rrp1	a
pREP81-FLAG	a
pREP81-Rrp1-FLAG	a
pREP81-Rrp1-DAEA-FLAG	a
pREP81-Rrp1-CS-FLAG	a
pREP81-Rrp2-FLAG	a
pREP42-EGFP	b
pREP42-EGFP-Rrp1	a
pREP42-EGFP-Rrp1-CS-FLAG	c
pREP42-EGFP-Rrp2	a
pREP41-Rad51	d
pREP41-Rad51-L1	d
pGADT7	a
pGADT7-Rrp1	a
pGADT7-Rrp2	a
pGADT7-Rrp1 (1-226)	c
pGADT7-Rrp1 (227-482)	c
pGADT7-Rrp1 (483-694)	c
pGADT7-Rrp1 (695-897)	c
pGADT7-Rrp2	a
pGADT7-Rad51N (1-117)	d
pGADT7-Rad51C (114-365)	d
pGBKT7	a
pGBKT7-Rrp1	a
pGBKT7-Rrp2	a
pGBKT7-Rad51N (1-117)	d
pGBKT7-Rad51C (114-365)	d
pYK788 pREP1- His_6-Ubi1	d

a - laboratory stock
b - Craven,R.A., Griffiths,D.J., Sheldrick,K.S., Randall,R.E., Hagan,I.M. and Carr,A.M. (1998) Gene, 221, 59–68.
c - this study
d - Hiroshi Iwasaki laboratory

Table S3 Primers used in this study:

Cloned gene/qPCR primers	Primer name	Primer sequence
FLAG	REP3flag_a_R	CTTTATCATCGTCGTCCTTGTAGTCGGATCCTCTAGAGTCGACATATGATTTAAC
	REP3flag_b_F	TACAAGGACGACGATGATAAAGACTACAAGGACGACGATGATAAAGACTA
	REP3flag_c_R	GGGTCATTTATCATCGTCGTCCTTGTAGTCTTTATCATCGTCGTCCTTGTAGT
	REP3flag_d_F	CAAGGACGACGATGATAAATGACCCGGGTAAAAGGAATGTCTCCCTTGCCAGTAC
Rrp1-FLAG	Rrp1_F	CAACTAATTATTCGAAACGGAATTCGAAACGATGGATTCATTGTCTGCATATC
	Rrp1_R	TTTAAATGGCCGGCCGGTACCTCATGAATTAAGCCCAAATAG
Rrp1-DAEA-FLAG (D397A; E398A)	Rrp1_F	CAACTAATTATTCGAAACGGAATTCGAAACGATGGATTCATTGTCTGCATATC
	Rrp1D397A_R	TATGTGCGGCCGCTAGAACAATGCGATAC
	Rrp1E398A_F	TGTTCTAGCGGCCGCACATACCATTCGT
	Rrp1E398A_R	TTTAAATGGCCGGCCGGTACCTCATGAATTAAGCCCAAATAGATATAG
Rrp1-CS-FLAG (C609S)	R1c609sN_F	TTTCTGACTTATAGTCGCTTTGTTAAATCATATGGATTCATTGTCTGCATATC
	R1c609sN_R	CAAACAAGGATCTAGACTAACACTACAGTTGAAATCC
	R1c609sC_F	AACTGTAGTGTTAGTCTAGATCCTTGTTTGGCTC
	R1c609sC_R	TAGTCTTTATCATCGTCGTCCTTGTAGTCGGATCCTGAATTAAGCCCAAATAGATATAG
Rrp2-FLAG	Rrp2_F	CAACTAATTATTCGAAACGGAATTCGAAACGATGAGAAATAATACAGCTTTTGAAC
	Rrp2_R	TTTAAATGGCCGGCCGGTACCTTATCGTGATGACATTCCAAATAAAAATG
Rrp1(1-226)	Rrp1_D1_F	ATAGTCGCTTTGTTAAATCATATGGATTCATTGTCTGCATATC
	Rrp1_D1_R	CATCGTCGTCCTTGTAGTCGGATCCGGGAGTATTATGCTGAAG
Rrp1(227-482)	Rrp1_D2_F	ATAGTCGCTTTGTTAAATCATATGAGTCCGTTCGACACGATC
	Rrp1_D2_R	CATCGTCGTCCTTGTAGTCGGATCCAGCCAAAAGGATGCGAAG
Rrp1(483-694)	Rrp1_D3_F	ATAGTCGCTTTGTTAAATCATATGTCTACGGTTTTTCGTAGAAC
	Rrp1_D3_R	CATCGTCGTCCTTGTAGTCGGATCCTTCTTGTTCTGAAAAAGATTG
Rrp1(695-897)	Rrp1_D4_F	ATAGTCGCTTTGTTAAATCATATGAGCATTAAATTAAGGTGGG
	Rrp1_D4_R	CATCGTCGTCCTTGTAGTCGGATCCTGAATTAAGCCCAAATAGATATAG
GST-Rrp1-FLAG, GST-Rrp1-CS-FLAG	Rrp1_F	GGGGCCCCTGGGATCTCATATGGATTCATTGTCTGCATATCC
	Rrp1_R	CTCGAGTCGACCCGGGGATCCGCAAGGGAGACATTCCTTTTACC
qPCR: RFB (RTS1-RFB locus)	L3_F	TTTAAATCAAATCTTCCATGCG
	L3_R	TGTACCCATGAGCAAACTGC
qPCR: cnt (core centromere)	cnt_F	GTATTAGTGGTCGGTTTTCTTTTTGTT
	cnt_R	CGGCGAAATGCTTCAGACAT
qPCR: dg (dg repeats)	dg_F	CGGTCTTTGCAGGACTCTTGA
	dg_R	CCACCACAATTTAACCCGATTAG
qPCR: tel (telomere)	tel_F	GTTACTCGCCTGCCTCTACCAT
	tel_R	GTCAGTCAAGTTAATGAGTCATGAAGAA
qPCR: ade6, Control locus on ChrIII	ade6-23	GGCTGCCTCTACCATCATTC
	ade6-25	TTAAGCTGAGCTGCCAAGGT
qPCR: II.50, Control locus on ChrII	II.50_F	CACCGCAGTTCTACGTATCCT
	II.50_R	CGATGTAACGGTATGCGGTA

Suplementary Figures

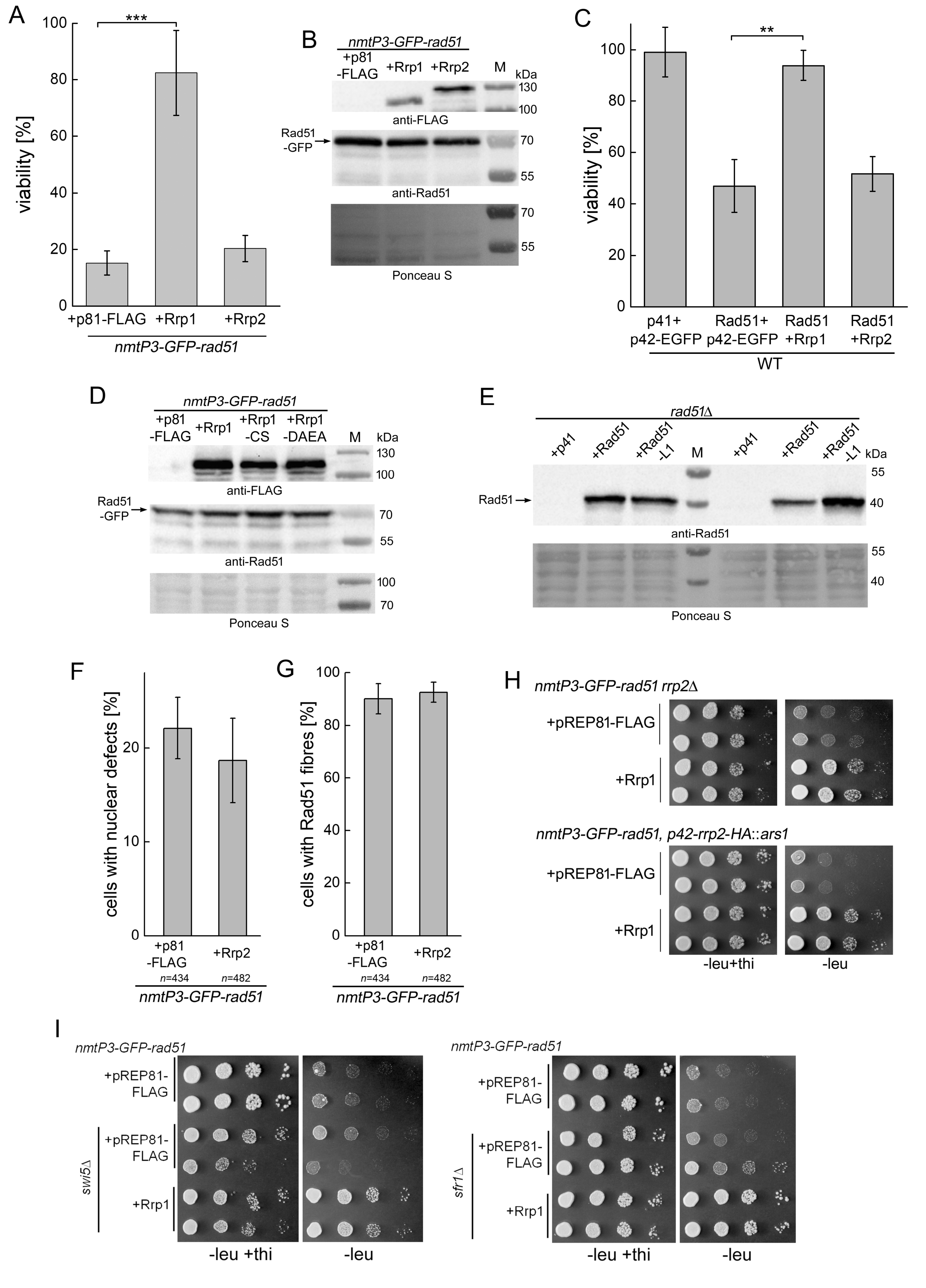

Figure S1 Involvement of other factors in the Rad51-Rrp1 interaction

Simultaneous overexpression of *rrp1*$^{+}$, but not *rrp2*$^{+}$, reverses viability loss in cells overexpressing *GFP-rad51*$^{+}$ (A), with both Rrp1 and Rrp2 produced at similar levels (B), or overexpressing *rad51*$^{+}$ (C), determined as ratio of surviving cells of indicated transformants

grown under inducing conditions to those grown without induction. The experiment was repeated with at least three independent transformants. Error bars represent the standard deviation about the mean values. ANOVA t-test was used to calculate *p*-values. (D) Rrp1, Rrp1-DAEA and Rrp1-CS protein levels were comparable as seen in whole cell extracts from transformants of the *nmtP3-GFP-rad51* strain probed with anti-FLAG antibodies. (E) Rad51-L1 was produced at similar levels to wild-type Rad51 from the constructs used in the study, as seen in whole cell extracts from *rad51*Δ strain transformed with plasmids harbouring genes for the indicated variants of Rad51, probed with anti-rad51 antiserum.Two sets of transformants are shown. (F) Over-expression of *rrp2*$^{+}$ does not reduce the number of *nmtP3-GFP-rad51* cells undergoing aberrant mitosis. Cells with unequally segregated genetic material (cut and non-disjunction) were observed by DAPI staining of the nuclei of transformants grown for 48 h in media lacking thiamine. Error bars represent the standard deviation about the mean values. n = total number of cells counted for 3 independent transformants for vector and *rrp2*$^{+}$. (G) Rad51 fibres are still present on chromatin in *nmtP3-GFP-rad51* cells overexpressing *rrp2*$^{+}$. n = total number of cells counted for 3 independent transformants for vector and *rrp2*$^{+}$ grown for 48 h in media lacking thiamine. Error bars represent the standard deviation about the mean values. (H) Deletion or overexpression of *rrp2*$^{+}$ does not affect suppression of the *nmtP3-GFP-rad51* growth defect by overproduction of Rrp1. (I) The rescue of the *nmtP3-GFP-rad51* growth defect by *rrp1*$^{+}$ overexpression is not dependent on the presence of *swi5*$^{+}$ or *sfr1*$^{+}$.

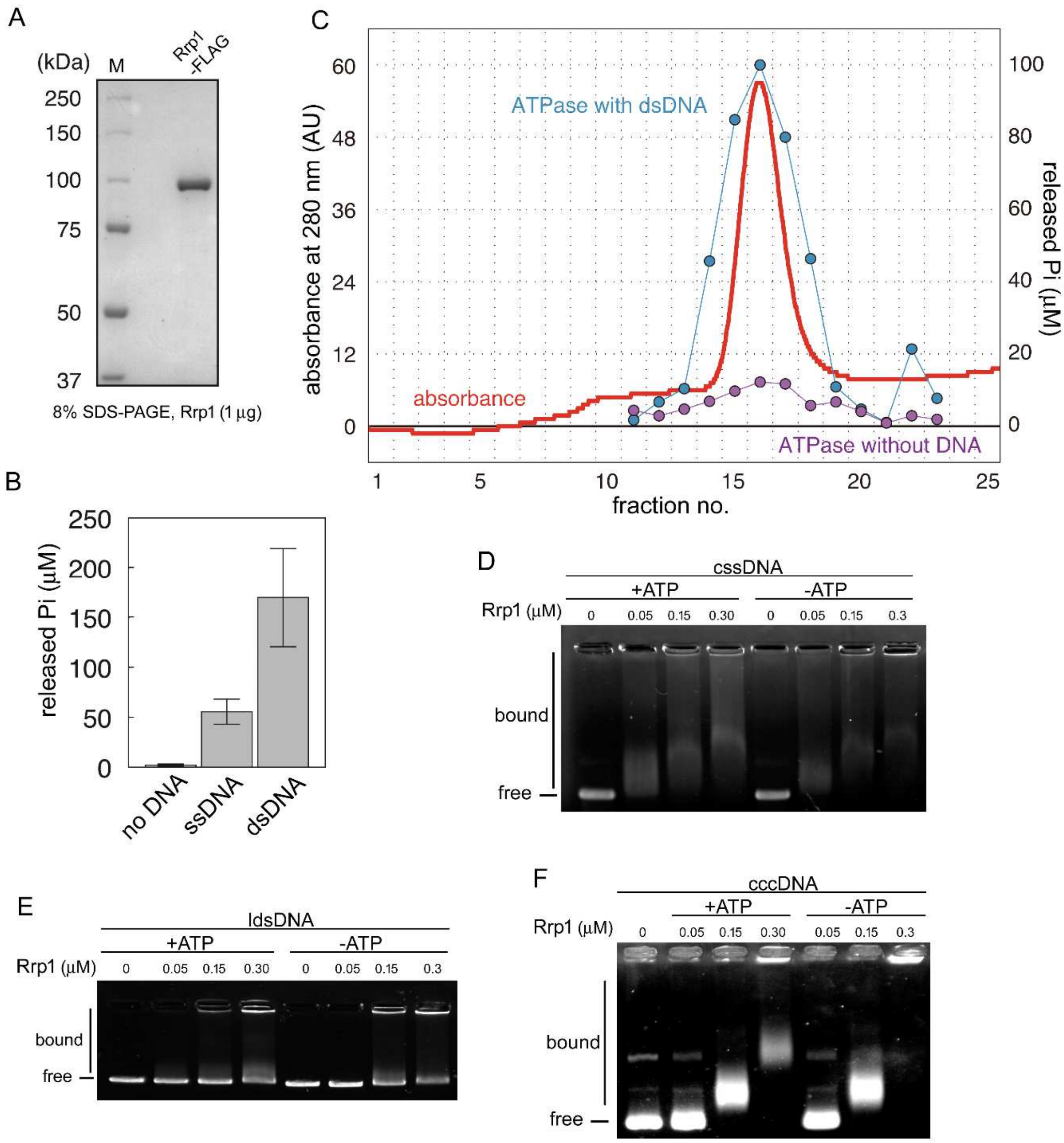

Figure S2 Purified Rrp1 possesses DNA-dependent ATPase activity and binds both ssDNA and dsDNA

(A) Purified recombinant Rrp1 protein was analysed by SDS-PAGE followed by Coomassie Brilliant Blue staining. (B) Rrp1 has a robust DNA-dependent ATPase activity. Purified Rrp1 (30 nM) was incubated with or without DNA substrates (5 µM nucleotides ssDNA or 5 µM base pair dsDNA) and 1 mM ATP. Measurements were done in triplicates for two independent protein preparations. (C) ATPase activity, measured as released Pi, and protein concentration, measured by absorbance at 280 nm, in the peak fractions from the last step of Rrp1 purification. (D-F) Electrophoretic mobility-shift assay (EMSA) demonstrating that Rrp1 binds to circular single-stranded DNA (cssDNA) (D), linear double-stranded DNA (ldsDNA) (E), and circular double-stranded DNA (cccDNA) (F) in an ATP-independent manner.

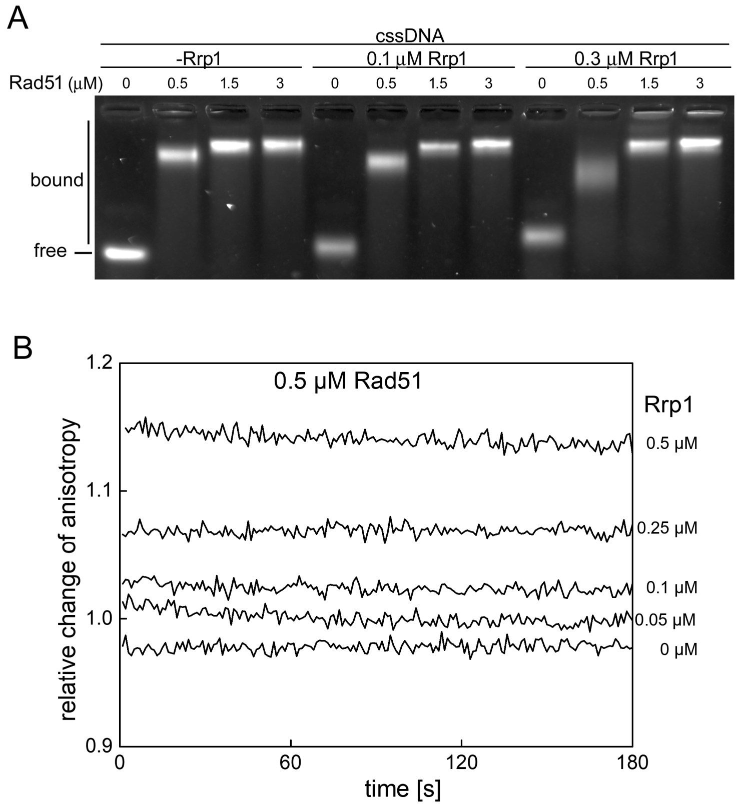

Figure S3 Rrp1 does not disassemble Rad51-ssDNA complexes

(A) Rrp1 has little effect on preformed Rad51-ssDNA filaments, as determined by EMSA. The indicated concentrations of Rad51 were incubated with cssDNA before the addition of Rrp1. (B) Rad51 filaments were formed on fluorescently labelled ssDNA, the indicated concentration of Rrp1 was added, and fluorescent anisotropy was measured in real-time. The addition of Rrp1 led to an increase, not a decrease, in relative anisotropy, indicating that Rrp1 does not disrupt Rad51-ssDNA complexes. The anisotropy increase observed in the presence of Rrp1is likely due to binding of Rrp1 to Rad51-ssDNA complexes.

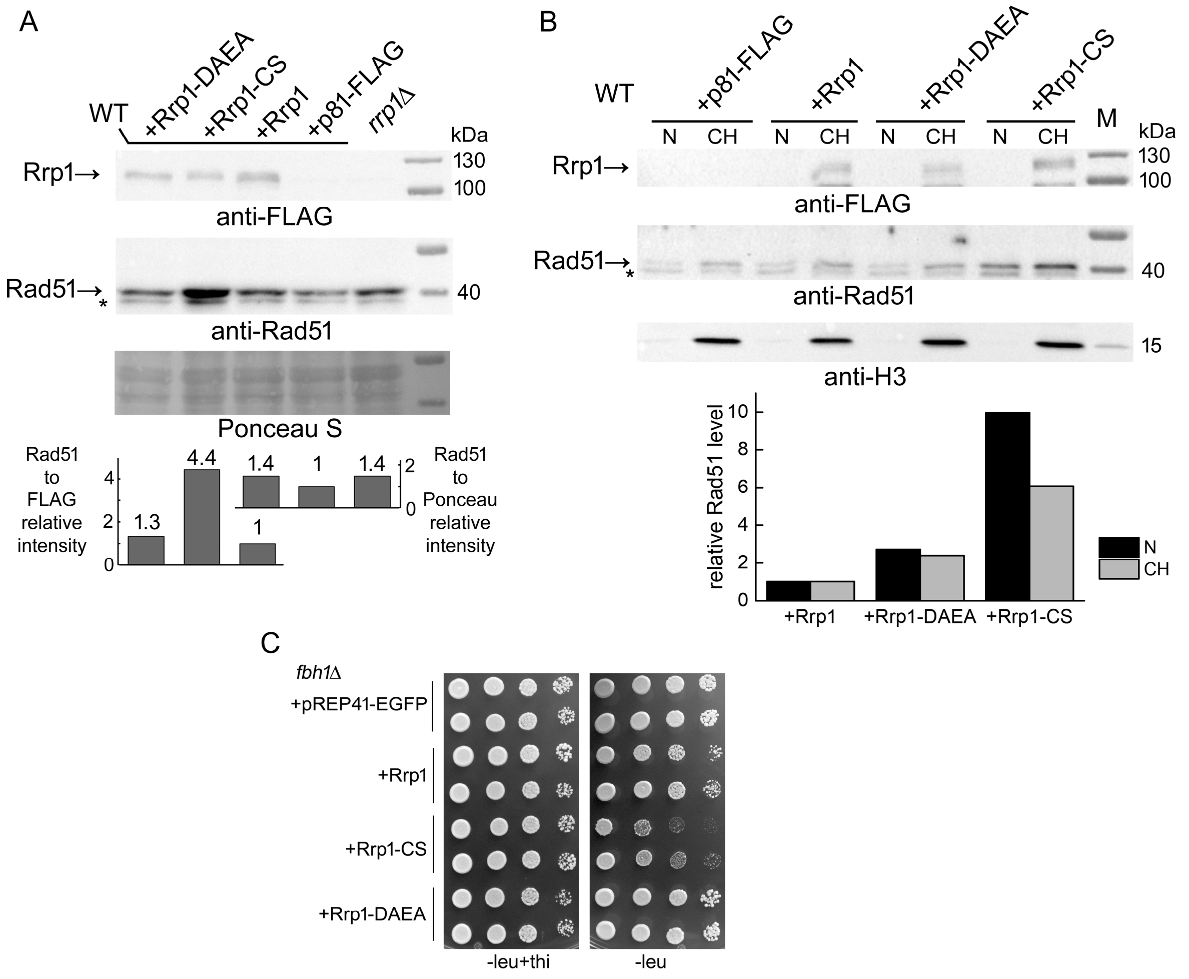

Figure S4 Rrp1 ubiquitin ligase activity has a role in Rad51 regulation *in vivo*

(A) Deletion or overexpression of *rrp1*$^{+}$ has no effect on native Rad51 levels, but overproduction of Rrp1-CS leads to accumulation of Rad51. Total protein extracts were isolated from respective cultures grown for 48h under expression inducing conditions and analysed by western blot with Rad51 antiserum and anti-FLAG antibody (for Rrp1). Experiment was repeated 3 times, data from representative blot were quantified and are shown as relative intensities of anti-Rad51 versus Ponceau loading control (examination of the effect of Rrp1 presence, right bar graph), where reads were normalised by the value obtained for vector control sample; and as relative intensities of anti-Rad51 versus anti-FLAG (examination of the influence of Rrp1 domains, left bar graph) where reads were normalised by the value obtained for WT Rrp1 overproducing sample. (B) Rad51 accumulates both in nucleoplasmic (N) and chromatin (CH) fractions in cells overproducing Rrp1-CS. Fractionation of proteins isolated from respective cultures grown for 48 h under expression inducing conditions and analysed by western blot probed with Rad51 antiserum, anti-FLAG antibody (for Rrp1), and anti-H3 antibody (histone H3, chromatin fraction control). Experiment was repeated 3 times, data from representative blot were quantified and are shown as relative intensities of signals for Rad51 versus FLAG (for Rrp1). (C) Overproduction of Rrp1 RING mutant (Rrp1-CS) is more toxic than WT Rrp1 in the *fbh1Δ* background. Cells were transformed with plasmids harbouring genes for the indicated variants of Rrp1 and the effect of their overexpression on growth (-leu) was assessed by spot test analysis. Two independent transformants for each type of plasmid are shown.
